# Endothelial dysfunction, platelet hyperactivity, hypertension, and the metabolic syndrome: molecular insights and combating strategies

**DOI:** 10.3389/fnut.2023.1221438

**Published:** 2023-08-08

**Authors:** Diptimayee Das, Nagainallur Ravichandran Shruthi, Antara Banerjee, Ganesan Jothimani, Asim K. Duttaroy, Surajit Pathak

**Affiliations:** ^1^Faculty of Allied Health Sciences, Chettinad Hospital and Research Institute, Chettinad Academy of Research and Education, Kelambakkam, Tamil Nadu, India; ^2^Faculty of Medicine, Department of Nutrition, Institute of Medical Sciences, University of Oslo, Oslo, Norway

**Keywords:** metabolic syndrome, atherosclerotic cardiovascular disease, hypertension, endothelial dysfunction, platelet hyperactivity

## Abstract

Metabolic syndrome (MetS) is a multifaceted condition that increases the possibility of developing atherosclerotic cardiovascular disease. MetS includes obesity, hypertension, dyslipidemia, hyperglycemia, endothelial dysfunction, and platelet hyperactivity. There is a concerning rise in the occurrence and frequency of MetS globally. The rising incidence and severity of MetS need a proactive, multipronged strategy for identifying and treating those affected. For many MetS patients, achieving recommended goals for healthy fat intake, blood pressure control, and blood glucose management may require a combination of medicine therapy, lifestyles, nutraceuticals, and others. However, it is essential to note that lifestyle modification should be the first-line therapy for MetS. In addition, MetS requires pharmacological, nutraceutical, or other interventions. This review aimed to bring together the etiology, molecular mechanisms, and dietary strategies to combat hypertension, endothelial dysfunction, and platelet dysfunction in individuals with MetS.

## 1. Introduction

Metabolic syndrome (MetS) is a medical condition characterized by a combination of metabolic abnormalities, including insulin resistance, hyperglycemia, hyperlipidemia, hypertension, and obesity. This condition has a negative impact on the vascular wall due to events involving endothelial dysfunction, platelet hyperactivity, oxidative stress, and low-grade inflammation. The activation of these events leads to increased vasoconstriction and atherosclerosis, ultimately promoting a pro-thrombotic state (1). Clinical studies have shown that endothelial dysfunction, hyperlipidemia, oxidative stress, and platelet hyperactivity are important factors in the development of atherosclerotic vascular problems (2). There is still a need for further improvement in our understanding of the molecular mechanisms underlying endothelial dysfunction, platelet hyperactivity, high blood pressure, and vascular damage. The challenge of comprehending the role of the vascular endothelium in the development of hypertension persists. A well-functioning endothelium is responsible for producing vasodilators that play a crucial role in maintaining healthy blood vessel function and vascularity. Endothelial dysfunction is a condition that is marked by a decrease in endothelial-dependent vasodilation and an increase in endothelial inflammatory activity (3). Endothelial dysfunction increases the likelihood of the blood vessels being in a more contracted state. This is caused by an imbalance between factors that relax the endothelium, such as nitric oxide (NO), prostacyclin (PGI_2_), and down regulation of endothelioum-dependent hyperpolarization (EDH) and factors that cause contraction such as thromboxane A_2_ (TxA_2_) and upregulation of endothelin-1 (ET-1). Endothelial dysfunction promotes pro-inflammatory and oxidative stress pathways through the generation of reactive oxygen species (ROS) in endothelial mitochondria. This, in turn, drives vascular growth and remodeling. The endothelium undergoes a significant transformation in metabolic syndrome, shifting to a dysfunctional state. This transformation involves the host immune system and the production of reactive oxygen species (ROS). The progression of diseases associated with metabolic syndrome occurs through a range of dynamic changes within the vasculature (4). Although there is a general consensus that endothelial dysfunction is a reliable indicator of the advancement of atherosclerosis and potential cardiovascular events, additional research is needed to fully understand its impact on hypertension and platelet hyperactivity. The components of MetS, whether alone or in combination, are significant risk factors for the development of cardiovascular diseases (CVDs). As a result, the implications for future healthcare costs and management are both relevant and challenging. Although underlying metabolic or genetic predispositions are essential, the condition typically affects individuals who consume high-caloric foods with sedentary lifestyles (5); modifying lifestyles, such as physical exercise and diet, can address many causative factors contributing to MetS. Diet modification represents an essential strategy for MetS prevention, as the increasing evidence indicates a robust inverse association between CVD risks, MetS, and the consumption of plant-based foods and their bioactive compounds.

This review highlights the prevalence, molecular mechanisms and combating approaches of underlying risk conditions by managing endothelial dysfunction, hypertension, and platelet hyperactivity/prothrombotic state with bioactive compounds like polyphenols, small molecules like miRNA, and gene therapy. The study also highlights the potential effects of lifestyle measures and pharmacological interventions on endothelial function in patients diagnosed with MetS.

### 1.1. Prevalence of MetS

MetS has a varying prevalence across the globe and is frequently associated with central obesity, diabetes, and CVD. Prevalence rates differ significantly based on age, gender, racial or ethnic background, and specific diagnostic criteria. MetS affects around 25% of the European population and at least 20% of the American people. MetS is less prevalent in South and Southeast Asia. However, its incidence has gradually increased and is expected to reach the levels observed in Western countries. With a 95% confidence range of 28 to 33%, 30% of Indian adults have MetS. According to research, one-third of people in low- and middle-income nations in the Middle East, Southeast Asia, and Latin America showed MetS symptoms (6). According to the study, India’s rapid economic development and urbanization may explain its rising MetS rate, and it may affect lifestyle and nutrition (7). According to study, MetS was more common in urban (32%; 95% CI: 29–36%) than tribal (28%; 21–36%) settings and it may contribute to urban MetS due to unfavorable lifestyle choices, higher socioeconomic class, lesser physical exercise, increased stress, and excessive salt and red meat consumption (8). MetS affects both genders and ethnic groups differently. The prevalence of MS was higher in females (35%; 95% CI: 31–38%) than in males (26%; 95% CI: 22–29%). Similar discoveries have also been made in the Eastern Mediterranean, South East Asia, and Western Pacific regions (9). This conclusion is attributed to factors such as menopause, polycystic ovarian syndrome, and the use of hormonal contraceptives (10). Additional factors contribute to the differences between men and women, such as the increased risk women face due to certain characteristics. These characteristics include higher body weight, larger waist circumference, and lower HDL cholesterol levels (11). MetS is more prevalent in Hispanic women by 26% compared to Hispanic men and in African-American women by 57% compared to African-American men. Insulin resistance is a predominant symptom of MetS among Hispanics, while African-Americans are more likely to experience hypertension, and dyslipidemia is more common among Whites (5). Miller and colleagues conducted a more recent analysis of the same database and discovered that 10.1% of adolescents in the United States had MetS (12). They demonstrated that MetS was more prevalent among men and Hispanics than women. The incidence of MetS rises significantly in tandem with an individual’s Body Mass Index (BMI). The prevalence of MetS is 9.8% among men in urban North India while 42.0% among women in urban Iran (13). The growth is evident irrespective of the parameters considered, and it indicates a shift from a conventional, older lifestyle to a contemporary, Western one. As developing countries grew economically more robust, they experienced a demographic transition. This transition led to a decrease in the prevalence of infectious diseases and an increase in the majority of lifestyle-related diseases. During this period, there was a shift in the prevalent types of conditions. Infectious diseases became less common, while lifestyle-related diseases became more prevalent. This change occurred alongside what is known as an epidemiological transition. Disruption of these processes can result in increased BMI, generalized and abdominal obesity, hypertension, dyslipidemia, type 2 diabetes, and CVD (13).

### 1.2. Etiology of MetS

Despite decades of investigation, the precise cause of MetS remains unclear. A poor diet, insufficient exercise, and the consequent weight gain are the primary causes of the condition in most instances (14). Other contributing variables include endothelial dysfunction, elevated blood pressure, and hyperactive platelets. As aforementioned, the main objective behind establishing the MetS was to recognize characteristics linked with a high susceptibility to CVD (15). The term “syndrome” suggests that the underlying cause of MetS is not readily identifiable, and it was not initially expected that a singular, comprehensive pathological etiology would exist for MetS (16). MetS is closely concomitant to a “Westernized lifestyle” that includes sedentary behavior and easy access to high-fat meals. Obesity in children is a risk factor for adult MetS (17). In addition, many features of MetS, including its potential significance, are more widespread among low-income people. Even though not everyone with MetS has them, hereditary variables for both the syndrome’s components and body fat and muscle are now well established (18). Approximately 30–40% of the reported variation in body mass index (BMI) and 70–80% in fat distribution is attributable to hereditary factors. In 2007, the first single nucleotide polymorphism (SNP) linking high BMI to the fat mass and obesity-related (FTO) gene was found (19). The FTO gene affects mood and metabolism, potentially contributing to obesity. Since then, researchers have used SNPs to identify about 40 genetic variants connected to body mass index, fat distribution, obesity risk, and MetS (20). Although common allelic alterations only account for 2% of the variation in obesity, those with a higher number of risk alleles (>10) acquire more body weight than those with a smaller number (21). Recent evidence suggests that environmental factors must interact with obesity’s genetic basis. Lifestyle variables that increase intra-abdominal fat and metabolic risk include being overweight, eating high-fat, smoking, physically inactive, and drinking excessively (22).

### 1.3. Pathophysiology of MetS

The MetS has a harmful influence on many bodily systems. Endothelial dysfunction, vascular resistance, hypertension, and arterial wall inflammation are more likely to occur in patients with pre-existing microvascular damage, such as that caused by insulin resistance (23). Endothelial dysfunction can disrupt homeostasis, increasing the risk of atherosclerotic disease and hypertension. Renal impairment, elevated vascular resistance and stiffness, and structural cardiac disorders like left ventricular hypertrophy and stroke are all brought on by the adverse effects of hypertension on the body’s normal functioning (24).

Ischemic heart disease is a problem that may develop as a result of the cumulative effects of MetS, such as endothelial dysfunction and hypertension. Endothelial dysfunction caused by elevated plasminogen activator type 1 and adipokines may lead to thrombosis. For instance, coronary artery disease is a possible outcome of hypertension and other conditions related to MetS. Hypertension can lead to coronary artery disease (CAD) due to increased vascular resistance. Dyslipidemia can cause symptomatic ischemic heart disease in some patients and is associated with MetS. This disorder has the potential to accelerate the advancement of atherosclerosis (25).

### 1.4. Clinical-based definition of MetS

Although MetS has several components and clinical implications, there is currently no universally accepted pathogenic mechanism or firmly established diagnostic criteria for this condition. Furthermore, it is unclear whether this entity represents a distinct medical condition or acts as a substitute for a combination of risk factors that contribute to an individual’s vulnerability to a particular ailment. The prevalence of MetS is on the rise among children and young adults. The emerging aspect of the disease is significant because it will have future consequences for the global health burden (26). The Adult Treatment Panel III report (ATP III) from the National Cholesterol Education Programme highlighted the significance of MetS as a multifaceted risk factor for CVD (27). The report indicated that this condition necessitates further therapeutic attention. MetS can be diagnosed using the National Cholesterol Education Programme Adult Treatment Panel III (NCEP ATP III) definition. The diagnostic criteria for MetS consist of five parameters. These parameters include a waist circumference that exceeds 40 inches in men or 35 inches in women, blood pressure that surpasses 130/85 mmHg, fasting triglyceride levels that exceed 150 mg/dL, fasting high-density lipoprotein (HDL) cholesterol levels below 40 mg/dL in men or 50 mg/dL in women, and fasting blood sugar levels that exceed 100 mg/dL (28).

#### 1.4.1. Insulin resistance

Insulin resistance plays a crucial role in developing and advancing CVD. It is a MetS component and is commonly linked to obesity and dyslipidemia. In addition, it has been linked to chronic low-grade inflammation. Insulin resistance is linked to several negative consequences, such as endothelial dysfunction, reduced cardiac diastolic relaxation, impaired vascular relaxation, decreased coronary blood flow, and increased susceptibility to ischemia.

The pathophysiology of MetS and its components, including elevated triglycerides, blood pressure, glucose, waist circumference, and low high-density lipoprotein (HDL) cholesterol, are closely linked to insulin resistance and abdominal obesity. MetS Consequently, this section aims to explore how insulin resistance and abdominal obesity may contribute to the pathophysiology of MetS (29). To explain the pathophysiology of MetS, the concept of insulin resistance has emerged as the most popular and unifying framework. The definition of insulin resistance has always centered on glucose (30). Lack of insulin action leading to fasting hyperinsulinemia to maintain euglycemia indicates insulin resistance. However, postprandial hyperinsulinemia may exist before fasting hyperinsulinemia develops (31). Insulin influences various biological activities, including amino acid absorption, protein synthesis and degradation, and triglyceride breakdown in adipose tissue.

Furthermore, it impacts the functioning of lipoprotein lipase and the release of VLDL cholesterol. The hormone insulin plays a crucial role in promoting the uptake of glucose in adipose and muscle tissue, as well as in synthesizing glycogen in muscle and secreting triglycerides (32). Therefore, an individual’s insulin sensitivity or resistance is frequently assessed by evaluating their reaction to glucose or insulin stimulation, whether administered orally or intravenously (33). One of the key reasons contributing to the development of insulin resistance is the presence of an excess of circulating fatty acids.

**Figure 1:**
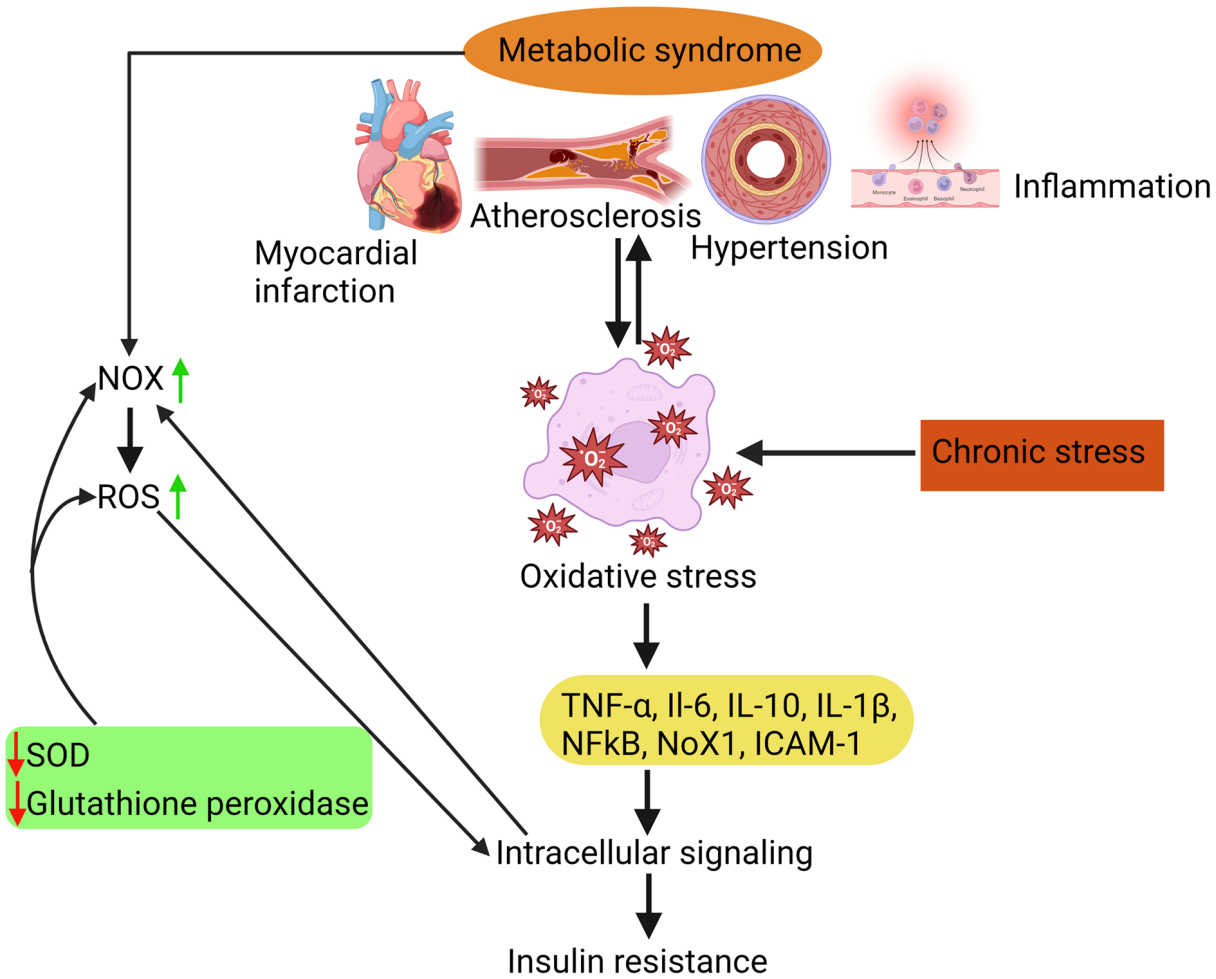
Insulin resistance due to oxidative stress is attributed to a disparity between the body’s antioxidant system and the generation of ROS, which in turn triggers inflammatory responses, atherosclerosis, hypertension, and myocardial infarction.

As illustrated in Figure 1, MetS is associated with an elevation in oxidative stress, which is the underlying cause of various complications such as atherosclerosis, hypertension, myocardial infarction, and inflammation. Elevated levels of oxidative stress have been identified as a detrimental factor contributing to insulin resistance development. This phenomenon occurs due to an imbalance between the body’s antioxidant defence mechanisms and the production of reactive oxygen species (ROS). β-cells that have low levels of antioxidants are especially susceptible to chronic oxidative stress. This stress can cause a decrease in the production of inflammatory cytokines like TNF-α and interleukins (such as IL-6, IL-10, and IL-1β), ultimately leading to the death of these cells.

### 1.5. Risk factors of MetS

**Figure 2:**
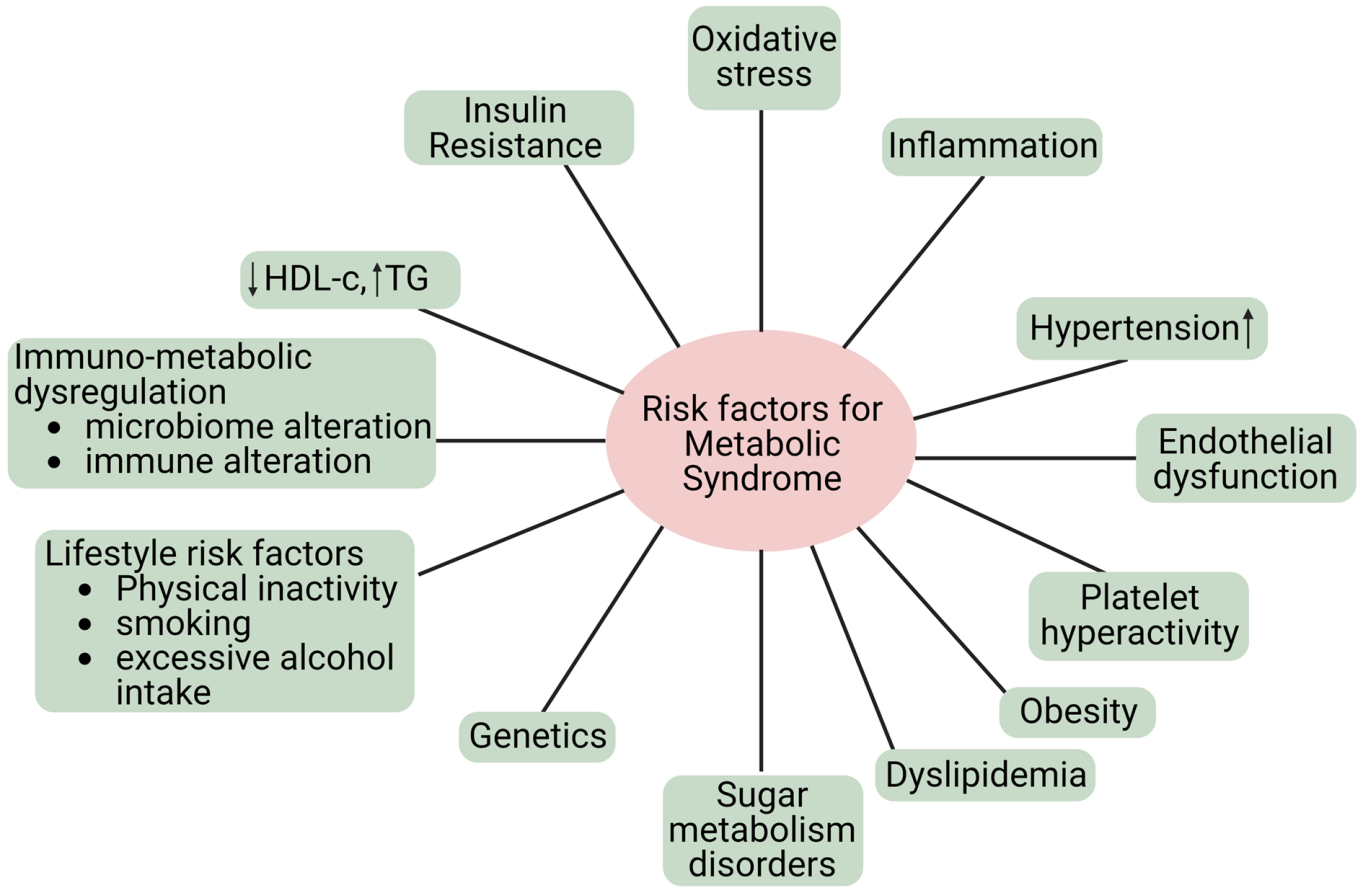
Risk factors of MetS.

MetS is a group of risk factors linked to developing atherosclerotic CVD and type 2 diabetes mellitus. Metabolic risk factors are the leading cause of noncommunicable diseases (NCDs) (34). Additionally, MetS refers to a group of metabolic anomalies linked to visceral fat accumulation. The disorders above encompass insulin resistance, hypertension, dyslipidemia (characterized by low levels of high-density lipoprotein cholesterol and hypertriglyceridemia), and central obesity (35), as shown in Figure 2. The primary causes of these metabolic risk factors include a poor diet, inactivity, an unhealthy lifestyle, smoking, alcohol consumption, etc. Hypertension, obesity, hyperglycemia, and hyperlipidemia are metabolic risk factors that increase NCD susceptibility (36).

### 1.6. Rationale

MetS is associated with insulin resistance, abdominal obesity, hyperlipidemia, hypertension, platelet hyperactivity, and other CVD risk factors. Individuals with MetS may require pharmacological and nutraceutical modifications to alleviate the unfavorable effects that lead to endothelial dysfunction, platelet hyperactivity, and hypertension. The proper functioning of the endothelium is crucial for maintaining homeostasis, and any disturbance in its function can lead to atherosclerosis, hypertension, and diabetes. Endothelium dysfunction is characterized by impaired endothelial cell-dependent vasodilation, inflammation, apoptosis, and cell proliferation. It is essential to comprehend and address endothelial dysfunction to prevent vascular complications.

## 2. MetS and cardiovascular disease

Each component of the MetS has been documented as a risk factor for CVDs. Moreover, the cumulative effect of multiple risk factors goes beyond simply adding up individual risks, further impacting the overall risk (37). In recent research, it has been found that MetS, which was defined by the World Health Organisation (WHO) in 1999, is strongly associated with an increased risk of coronary heart disease (CHD). These individuals were at a higher risk of experiencing myocardial infarction and cerebrovascular disease. The disorder was also found to be associated with a significant increase in cardiovascular mortality (38). Having diabetes significantly increases the likelihood of developing CVD in individuals with MetS. Many individuals with type 2 diabetes, particularly those who also have other risk factors for cardiovascular disease, face a substantial risk of experiencing severe cardiovascular events (39). In a study involving a large group of individuals with type 2 diabetes, insulin resistance (IR) was found to be a significant factor in predicting both the prevalence of CVD at the beginning of the study and the occurrence of new cases of CVD during the follow-up period. There was no evidence to suggest a direct relationship between smoking, variables related to insulin resistance (IR), such as body mass index (BMI), and the subject in question. The researchers utilized data from the Kuopio Ischaemic Heart Disease Risk Factor study to examine the relationship between MetS and cardiovascular mortality (40). Even in the absence of CVD and diabetes at the beginning of the study, MetS was found to be linked to an increased risk of cardiovascular disease and cardiovascular mortality in males. Among individuals with MetS, the relative risks for mortality due to CHD and CVD were approximately four and three and a half, respectively (41).

## 3. MetS and diabetes

The worldwide prevalence of MetS and type 2 diabetes is increasing. Diabetes and MetS are associated with numerous health problems, such as hypertension, stroke, breast, prostate, and colon cancers, limb amputation, blindness, and renal and gallstone disease (42). Consequentially, the burden of chronic diseases on healthcare systems has been burdensome for Western nations and may be fatal for countries with limited resources (43). There is currently no cure for diabetes. Consequently, primary prevention via dietary and lifestyle modifications is crucial (44).

## 4. Management of MetS risk factors

The metabolic risk variables in the syndrome’s definition include atherogenic dyslipidemia, elevated blood pressure, high plasma glucose, a prothrombotic condition, and a pro-inflammatory state (45). If the underlying risk factors can be adequately addressed, the severity of all metabolic risk factors will be reduced. The most successful treatment for those with MetS should focus on promoting modest weight reduction and regular involvement in physically active leisure activities (46). The therapy must target the multi-pathological process involved with MetS, with each disorder component being recognized and aggressively treated (47). If lifestyle changes are insufficient, a multimodal treatment strategy will be needed to achieve the requisite blood pressure, lipid profile, and blood glucose control targets. In addition, clinical risk factors such as dyslipidemia, hypertension, and hyperglycemia should be addressed more aggressively. According to the professional agreement, appropriate LDL cholesterol or blood pressure objectives have yet to be determined in treating MetS (48).

Reduction in blood pressure has been demonstrated to reduce the risk of severe CVD in several clinical studies, including those involving people with type-2 diabetes (49). However, the most effective drug has yet to be fully resolved (50). Some studies have shown that angiotensin-converting enzyme (ACE) inhibitors or angiotensin receptor blockers are better first-line therapy for patients with MetS, especially when type-2 diabetes is present.

A prothrombotic state is indicated by elevated levels of fibrinogen, plasminogen activator inhibitor-1, and other coagulation components. Low-dose aspirin or other antiplatelet drugs are available to diabetic patients as a potential therapeutic option for managing the increased risk of arterial thrombosis (51). It is generally recommended that individuals with CVD take these medications, unless otherwise specified. Although clinical trials have not demonstrated the effectiveness of these drugs in individuals with type-2 diabetes who do not have CVD, they are still widely recommended (52). Furthermore, aspirin prophylaxis is a viable treatment option for individuals diagnosed with MetS who are at a relatively high risk of experiencing CVD-related events (53).

## 5. Role of MetS on endothelial dysfunction

The role of endothelial dysfunction in the development of atherosclerosis is significant. Prospective studies have indicated that endothelial dysfunction is a predictive factor for the occurrence of vascular events, regardless of the presence or absence of preexisting vascular disease. The involvement of endothelial dysfunction in developing T2DM has been observed. Due to the detrimental effects of all constituent elements of MetS on the endothelium, it is plausible that endothelial dysfunction is more prevalent among individuals with MetS. This dysfunction, in turn, may play a role in the increased vulnerability to vascular disease and T2DM observed within this particular cohort. There is ample documentation indicating that endothelial dysfunction, which is a response to cardiovascular risk factors, typically precedes the development of atherosclerosis (54). The treatment of cardiovascular risk factors leads to an improvement in endothelial function. Its ability to independently predict cardiac events is highly valuable. In addition to the elevated blood pressure readings obtained from the sphygmomanometer, patients with hypertension often experience other issues such as endothelial dysfunction, increased peripheral vascular resistance, and arterial stiffness. These problems can be detected in patients with hypertension. Metabolic syndrome is a condition characterized by the presence of abdominal obesity, insulin resistance, hypertension, dyslipidemia, and glucose intolerance. It is often considered a prediabetic condition. Metabolic syndrome frequently involves endothelial dysfunction (55). A research study discovered that within a sample of 100 male and female volunteers who were not diagnosed with diabetes and had no prior history of cardiovascular disease, individuals who met the ATP-III criteria for MetS exhibited a higher degree of endothelial dysfunction than those who did not. This finding is particularly significant because it was observed in individuals within an age range that is typically not associated with vascular disease. The level of endothelial dysfunction was strongly associated with the amount of metabolic components of the ATP-III criterion present in each participant. In a multivariate logistic regression model, it was found that the presence or absence of MS was the only independent predictor of endothelial dysfunction (55).The occurrence of endothelial dysfunction represents an initial pathogenic occurrence in the development of MetS. Increased cardiovascular morbidity and death are associated with this impairment (56). Endothelial coronary, peripheral, or cerebral vasculature dysfunction predicts vascular events. Endothelial dysfunction is linked to the pathophysiology of CVDs (57). The state of endothelial dysfunction is distinguished by a transition toward a pro-inflammatory state to diminished vasodilation and prothrombotic requirements, as well as a reduced ability to endure oxidative stress. This change may also be seen as a condition where thrombotic events are more prone to occur. These events create vascular inflammation, partly mediated by activated mononuclear cells’ ROS (58).

### 5.1. Endothelial dysfunction

Vascular homeostasis relies on the endothelium, a monocellular lining that coats the inside arteries and separates the interstitial fluid from the blood (59). Nitric oxide (NO), natriuretic peptide type C (NPyC), and eicosanoids are all paracrine chemicals usually secreted by the endothelium. The variables mentioned above impact the proportion of vasodilation to vasoconstriction, the movement and growth of smooth muscle cells, the hindrance and encouragement of platelet adhesion and aggregation, and thrombogenesis and fibrinolysis (60). Endothelial dysfunction is a significant risk factor for CVD and a precursor to atherosclerosis (61). Diabetes, peripheral vascular disease, stroke, and heart diseases (62) are only some of the vascular and metabolic disorders associated with oxidative stress and endothelial dysfunction in humans.

#### 5.1.1. Prevalence, prediction, and geographical status of endothelial dysfunction

MetS affects a significant portion of the population in different world regions. In the United States, it affects approximately 34.6% of the people, while in Europe, the prevalence ranges from 17.8 to 34.0%. In Asia, the prevalence ranges from 12.8 to 41.1% (63). The occurrence of endothelial dysfunction differed in different studies, ranging from 33 to 58%. The measurement of endothelial activation involves monitoring the levels of certain adhesion molecules in the blood, such as soluble intercellular adhesion molecule-1 (sICAM-1), soluble vascular cell adhesion molecule-1 (sVCAM-1), and E-selectin. Additionally, serum levels of von Willebrand factor (vWF), tissue plasminogen activator (tPA), and plasminogen activator inhibitor-1 (PAI-1) are also monitored. Furthermore, the presence of circulating mature endothelial cells, endothelial progenitor cells, and endothelial microparticles is taken into account (64). However, it is important to note that not all of these indicators are exclusive to endothelium dysfunction. Levels of adhesion molecules in the blood increase during inflammatory situations. Additionally, levels of PAI-1 are associated with insulin resistance, a significant characteristic of MetS (65). It has been suggested that microalbuminuria is an indicator of generalized endothelial dysfunction (66). Indicators of endothelial dysfunction, such as sICAM-1, tPA antigen, and PAI-1 levels and activity, are higher in individuals with Metabolic Syndrome (MetS) (67). Additionally, research has shown that as the number of components of Metabolic Syndrome (MetS) increases, there is a corresponding increase in both plasma PAI-1 levels and activity. Patients with MetS also have higher levels of vasoconstrictor endothelin-1 in their blood (68).The prevalence of microalbuminuria is higher in patients with Metabolic Syndrome (MetS) (69). Additionally, the occurrence of microalbuminuria increases as the number of MetS components rises. The multivariate analysis found that the significant predictors of ED were the presence of CAD, diabetes, cigarette smoking, and the overall number of CVD risk variables (70). According to (71) ED was found in 67% of individuals with three CVD risk factors but did not have coronary artery disease (CAD). Among 137 patients with CAD, 44% had a notable endothelial dysfunction characterized by vasoconstriction greater than 20% (71). According to a study, even individuals with very low cardiovascular risk, such as healthy men and women, have been observed to experience endothelial dysfunction. Passing specific percentiles in males aged 50–55 and females aged 70–75 indicate an increased risk of CVD. Men appear to have a higher risk compared to women (72).

### 5.2. Oxidative stress and endothelial dysfunction

Many disorders, such as hypertension, atherosclerosis, dyslipidemia, diabetes mellitus, CVD, renal failure, and ischemia–reperfusion damage, are primarily caused by endothelial dysfunction and oxidative stress. Several clinical circumstances involve the oxidation of tetrahydrobiopterin (BH4) and mitochondrial electron transport, as well as the inactivation of nicotinamide-adenine dinucleotide phosphate (NADPH) oxidase, xanthine oxidase (XO), cyclooxygenase, and uncoupled endothelial nitric oxide synthase (eNOS) (73). An imbalance that results in reduced NO production or increased ROS creation promotes endothelial dysfunction. Remodeling, platelet aggregation, vasodilation loss, inflammation, and smooth muscle cell proliferation may occur from this imbalance (74). Endothelial dysfunction is related to oxidative stress in cardiovascular disorders such as CAD and stroke (75). Renovascular hypertension, for example, is produced by constriction of the blood vessels, which activates the renin-angiotensin system and raises the synthesis of angiotensin II (Ang II), the system’s main active peptide, thus increasing ROS (76).

### 5.3. Epigenetics in endothelial dysfunction

The importance of epigenetics in developing endothelial dysfunction and CVD is becoming more evident. It plays a crucial role in regulating various aspects of these conditions, from their underlying mechanisms to potential treatments. An epigenetic study was conducted on CVD due to its significance in inflammation and vascular involvement. The study revealed several alterations that affect the course of CVD. Furthermore, epigenetics play a role in influencing risk factors for CVD, including but not limited to age, hypertension, smoking, excessive alcohol consumption, and diabetes. Epigenetic modifications refer to all heritable changes in gene regulation that do not involve alterations to the DNA sequence (77). The principal epigenetic mechanisms observed in human cells encompass DNA methylation, posttranslational modifications of histones, and the involvement of non-coding RNA molecules, including microRNAs and long non-coding RNAs. Evidence suggests that epigenetic mechanisms, including DNA methylation, histone modification, and microRNAs, impact the endothelium’s ability to respond to blood flow (78). There is mounting evidence that epigenetic processes such as NOS3 (eNOS) play a significant role in regulating crucial endothelial cell genes and are responsive to a wide range of intrinsic and environmental stimuli, including those involved in the etiology of CVD. The role of non-coding RNAs in histone protein modification and regulation in endothelial cell homeostasis and dysfunction is well established (79).

### 5.4. Molecular mechanisms

Two major pathophysiological causes of many diseases are endothelial dysfunction and oxidative stress. The endothelium also generates a variety of vasodilators and vasoconstrictors, including thromboxane A_2_, NO, endothelin, and Ang II (80). Endothelial dysfunction is distinguished by reduced NO bioavailability and reduced vasodilation connected to a proinflammatory and prothrombotic condition. This results in a mild and reversible disorder in the endothelium that affects the whole body. The endothelium also generates a variety of vasodilators and vasoconstrictors, including thromboxane A2, endothelin, and Ang II (80). The condition of endothelial dysfunction is marked by impaired vasodilation and a state of prothrombotic and proinflammatory activity. According to the literature, endothelial dysfunction impacts physiological processes such as LDL oxidation, vascular smooth muscle cell proliferation and migration, cell permeability, leukocyte adhesion, and platelet activation (81). In addition, more mitochondria-produced ROS accumulate when inflammation causes oxidative stress. Oxidation of macromolecules is triggered by excess ROS, which in turn triggers cell death by releasing cytochrome c (Cyt-c) (82). In addition to promoting leukocyte adherence and altering endothelial signal transmission and redox-regulated transcription factors, oxidative stress also raises vascular permeability. Recent research has linked endothelial dysfunction to dysregulation of non-coding RNAs (miRNAs, lncRNA) and alterations in gene regulatory networks (82).

**Figure 3:**
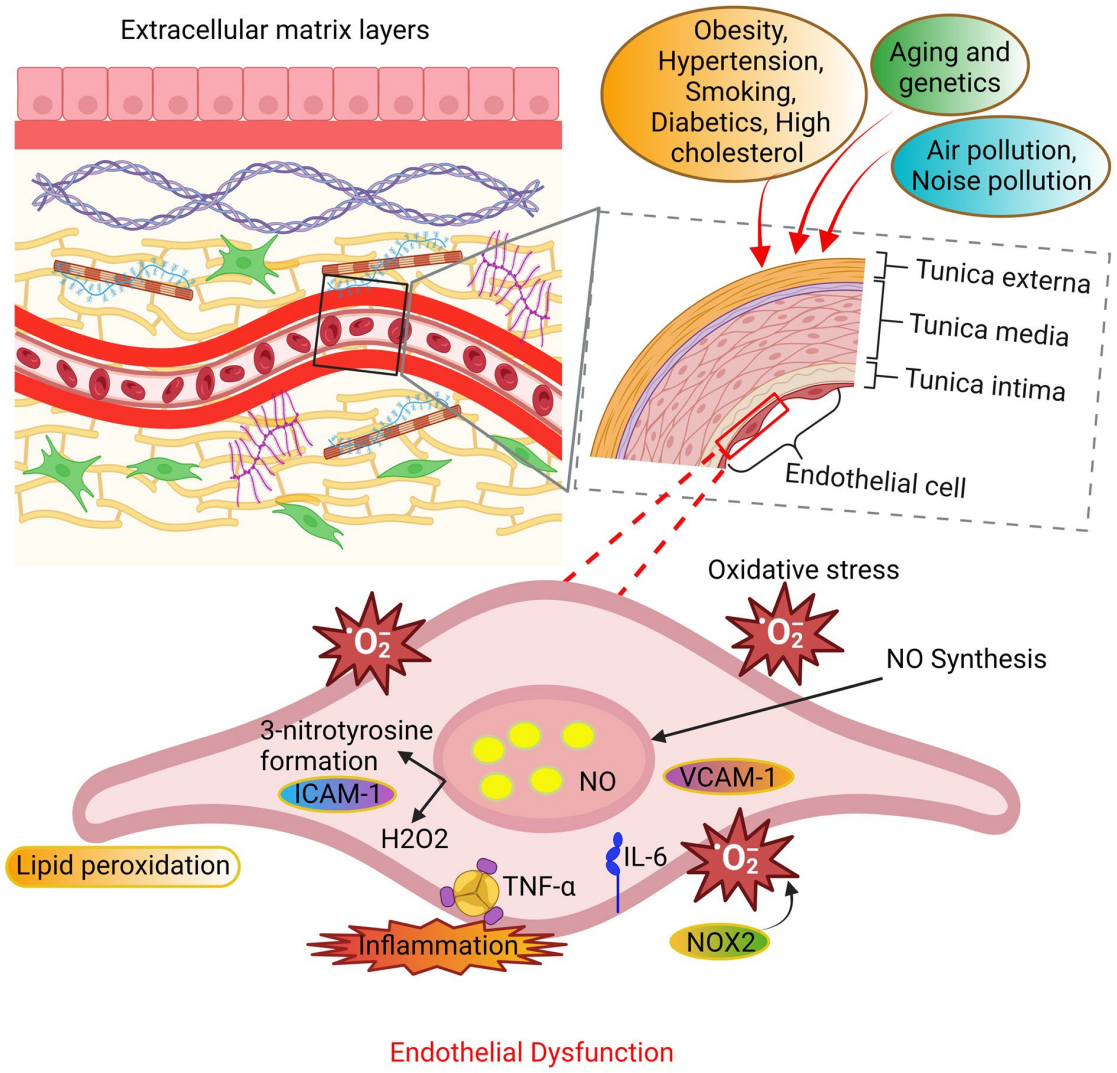
The mechanisms underlying endothelial dysfunction. The endothelial cells undergo oxidative stress due to alterations in their extracellular matrix layers caused by various factors such as aging, genetic predisposition, and environmental variables. The principal sources of ROS are NADPH oxidase and uncoupled nitric oxide synthase (NOS). The occurrence of endothelial function is attributed to an elevation in ROS beyond the innate antioxidant capabilities of the body. In addition, the production of substantial amounts of hydrogen peroxide (H_2_O_2_) contributes to the impairment of endothelial function and consequent cellular death.

Endothelial dysfunction, a recognized phenomenon, exerts an influence on the permeability of the endothelial barrier, a pivotal component involved in the inflammatory response that plays a contributory role in the initiation of CVD. Blood arteries are composed of various components, including connective tissue, endothelial cells, fibroblasts, and vascular smooth muscle cells (VSMCs). The endothelium, a semipermeable layer, serves as a barrier between the bloodstream and the innermost lining of blood vessels. As per Rahimi’s (83) findings, it has been observed that the endothelium possesses tightly specialized cell-to-cell junctions, which function as a selective barrier to impede the passage of macromolecules. Endothelial cells that have been stimulated and are in an activated state have the ability to generate various signalling molecules such as cytokines, chemokines, and growth factors. These molecules play a crucial role in enhancing the processes of endothelial cell proliferation, migration, and permeability (84). In accordance with the findings of Sun et al. (85), the introduction of endothelial cells with an inflammatory phenotype into the bloodstream’s arterial vessels results in the initiation of an inflammatory response, thereby contributing to the advancement of CVD. Based on the proposed theory, it is postulated that the primary etiological factor underlying hypertension, ageing, atherosclerosis, stroke, venous thrombosis, obesity, heart disease, diabetes, and intimal hyperplasia is the inflammatory response exhibited by endothelial cells (85). The loss of nitric oxide (NO) bioactivity is a common characteristic observed in the majority of individuals with cardiovascular risk factors. Consequently, this phenomenon holds significant therapeutic relevance (86). There is a discernible correlation between MetS, endothelial dysfunction, and CVD. While the significance of endothelial dysfunction in the onset of atherosclerosis is widely recognized, its specific contribution to the pathogenesis of CVD has yet to be definitively determined (Figure 3).

In type 2 diabetes patients, elevated blood sugar levels cause the vascular endothelium to become glycosylated. It modifies the blood vessels by making them narrower and more susceptible to rupture due to sprouting neovasculature (87). This leads to endothelial dysfunction from augmented ROS production, inflammation, and eNOS deletion (88). As a result, type 2 diabetes patients exhibit an increased susceptibility to CVD, with a two to four times greater risk than non-diabetic counterparts.

### 5.5. Endothelial dysfunction and diabetes

The presence of endothelial dysfunction has been observed to be closely linked with the occurrence of type 2 diabetes in human subjects. Endothelial dysfunction has been found to exhibit associations with various disorders related to diabetes, such as obesity, sedentary lifestyle, and MetS (89). Diabetes is characterized by elevated concentrations of endothelium-derived adhesion molecules, specifically ICAM-1, ICAM-2, VCAM-1, and PECAM-1, which are members of the immunoglobulin-like molecule family located on the surface of endothelial cells. In order to facilitate robust adhesion and/or transendothelial migration, these molecules interact with counter-receptors present on leukocytes and plasminogen activator inhibitor-1 within the circulatory system, indicating the potential presence of an endothelial phenotype that is both pro-inflammatory and pro-thrombotic (90). Multiple studies have been conducted, which have consistently demonstrated a higher prevalence of endothelial dysfunction among individuals diagnosed with diabetes. It is widely acknowledged within the scientific community that elevated blood glucose levels and the presence of diabetes have a detrimental effect on the production and functioning of NO. The available evidence indicates that there is a notable impairment in endothelial-derived nitric oxide (NO)-mediated vasodilation in individuals with both insulin-dependent and non-insulin-dependent diabetes mellitus (91).

### 5.6. Endothelial dysfunction and cardiovascular disease

The maintenance of a healthy endothelium is of utmost importance in the regulation of various cardiovascular functions, including but not limited to blood flow and fibrinolysis, hemostasis, vascular tone, angiogenesis, monocyte/leukocyte adhesion, and platelet aggregation. The aforementioned functions play a crucial role in the regulation of cardiovascular homeostasis, as indicated by previous research (92). The regulatory role of the vascular endothelium in maintaining cardiovascular health is widely recognized in scientific literature. The role of the aberrant vascular endothelium in the development of cardiovascular pathologies, including atherosclerosis, ageing, hypertension, obesity, and diabetes, has been widely recognized (93). Endothelial dysfunction, which refers to an imbalance between various factors involved in relaxation and contraction, coagulation and anticoagulation, as well as inflammation and anti-inflammation, may play a substantial role in the pathogenesis of atherosclerosis and cardiovascular disease (94). The presence of endothelial dysfunction has been observed in individuals diagnosed with ischemic CAD who also exhibit signs of atherosclerosis. Atherosclerosis is a pathological state that is distinguished by a deviation from the typical physiological behavior of endothelial cells. Specifically, these cells exhibit impaired release of nitric oxide in response to serotonin (95). The occurrence of vasoconstriction within atherosclerotic regions has the potential to instigate the progression of coronary thrombosis. It has been observed that there is a correlation between inflammation and CAD, wherein inflammation has been found to impede the production of endothelial nitric oxide. Patients diagnosed with CAD who experience myocardial ischemia are also at risk of mortality resulting from congestive heart failure (CHF) as a consequence of endothelial dysfunctions (96).

### 5.7. Treatment

Experimental, clinical, and translational evidence suggests that several medicines and therapeutic options may improve various aspects of endothelial dysfunction. Furthermore, most of these medications have shown encouraging cardioprotective benefits in preclinical and clinical research. Metformin reduced the risk of MetS and T2DM in overweight impaired fasting glucose (IFG) or impaired glucose tolerance (IGT) patients, although lifestyle adjustments were more beneficial. Metformin did not reduce MetS prevalence. Metformin decreased EDV in MetS patients with normal glucose tolerance independent of glucose, LDL-C, HDL-C, body weight, or blood pressure. In another MetS study, metformin improved Flow-Mediated Dilation (FMD) and insulin resistance. MetS patients’ NO levels also increased with metformin (97).

In the Diabetes Reduction Assessment with Ramipril and Rosiglitazone Medication (DREAM) research, rosiglitazone reduced T2DM and improved normoglycemia in IFG patients. Vascular events remained stable, and heart failure risk increased. In MetS studies, rosiglitazone improved FMD. Endothelial function improved with lower hsCRP and higher adiponectin. Pioglitazone reduced hsCRP in MetS patients, however endothelial function was not examined (98).

After the bezafibrate infarction prevention (BIP) study, a subgroup analysis found that bezafibrate lowers myocardial infarction (MI) in CHD patients. Bezafibrate reduces T2DM in obese people and those with impaired fasting glucose (IFG). Fenofibrate lowered MetS. In MetS patients, fenofibrate raised flow-mediated dilation (FMD) and decreased sICAM-1 and sVCAM-1. Bezafibrate increased FMD. Statins and fibrates together improve FMD in mixed dyslipidemia patients. When MetS patients moved from atorvastatin to bezafibrate, endothelial function decreased (99).

Nicotinic acid (NA) may treat MetS-related mixed dyslipidemia. A Coronary Drug Project study shows that NA reduces non-fatal MI and all-cause mortality in CHD and MetS patients. MetS patients had higher FMD. In MetS patients, ezetimibe with atorvastatin at 10 mg daily reduced LDL-C and improved Endothelium-dependent vasodilation (EDV) compared to 40 mg alone. In another experiment, ezetimibe + simvastatin 10 mg/day prevented a reduction in FMD after a high-fat meal better than simvastatin 80 mg/day alone. Eicosapentaenoic acid lowered plasma sICAM-1 and sVCAM-1 in MetS patients. Orlistat improved T2DM in obese people with IGT. Orlistat and sibutramine improved FMD in obese people. Orlistat and sibutramine also lower MetS. It is unknown how these drugs affect endothelial function in MetS patients (100).

Estradiol increases NO generation and endothelial function. Estradiol interacting with HDL particles is unexpected. Other HDL-associated estradiol esters may boost HDL’s atheroprotective activities, including macrophage cholesterol export. According to research, oral hormone therapy increases adhesion molecules, PAI-1, and tPA better than transdermal hormone therapy. The earlier intervention raised hsCRP more than the latter. Oral estradiol reduced E-selectin levels in postmenopausal women with MetS in trial w108. Transdermal estradiol had no effect. Estradiol increases NO and endothelial function. Estradiol seems to work through HDL particles. HDL-associated estradiol esters increase macrophage cholesterol efflux and other atheroprotective actions. According to, oral hormone treatment increases bloodstream adhesion molecules, PAI-1, and tPA more than transdermal hormone therapy,. The former increased hsCRP more than the latter. Oral estradiol lowered E-selectin levels in postmenopausal women with MetS, while transdermal did not (101).

#### 5.7.1. Lipid lowering-statin

Subgroup analysis of primary and secondary preventative trials found statins reduce vascular events in MetS patients. Statins reduced MetS risk in multiple trials. Atorvastatin reduced MetS prevalence, increased FMD, and decreased sICAM-1 (102). Pravastatin did not affect sICAM-1, sVCAM-1, or E-selectin levels in MetS patients, whereas simvastatin lowered PAI-1 activity. Statins may affect endothelial function differently in MetS patients, according to data. No comparative studies have been done to assess whether statin improves endothelial function better. Statins, which work by inhibiting the enzyme hydroxymethylglutaryl coenzyme A reductase, are the standard treatment for lowering high cholesterol levels. However, the effects of statins are not limited to decreasing lipid levels; they also have extra cholesterol-dependent or pleiotropic effects (103). In addition, statins have been shown to have cardioprotective benefits by reducing oxidative stress, enhancing endothelial function and inflammation, stabilizing susceptible plaques, and clot formation (103).

#### 5.7.2. Antihypertensive drugs

According to secondary prevention subgroup analyses, angiotensin-converting enzyme inhibitors (ACE-I) reduce vascular events in MetS patients. ACE inhibitors and angiotensin receptor blockers (ARBs) diminish the risk of T2DM in hypertensives. Diuretics and beta-blockers enhance T2DM risk. Calcium channel blockers (CCBs) are ineffective. In the placebo-controlled DREAM trial, Ramipril did not decrease T2DM or vascular disease in IFG patients. Ramipril-treated individuals had more normoglycemia relapses. A recent subgroup analysis of the Antihypertensive and Lipid-Lowering medication to Prevent Heart Attack Trial (ALLHAT) indicated that chlorthalidone medication increased the risk of T2DM but decreased the risk of vascular events compared to lisinopril. Compared to chlorthalidone, lisinopril increased systolic blood pressure throughout follow-up. MetS patients treated with amlodipine, chlorthalidone, or lisinopril had comparable risks of developing T2DM and vascular events. Irbesartan improved endothelial function in MetS in a study. Despite lowering blood pressure, irbesartan improved FMD and plasma PAI-1 levels. Statins or fibrates plus ACE-I or ARB improve FMD better than alone (104). ARBs, CCBs, and ACEIs have been shown to potentially improve endothelial function in individuals diagnosed with CVD (104). The aforementioned outcomes are accomplished through a range of mechanisms, including upregulating the expression of endothelial nitric oxide synthase (eNOS), enhancing the phosphorylation of eNOS at Ser1177, downregulating the expression of NADPH oxidase (NOX), increasing the levels of tetrahydrobiopterin (BH4) in the vasculature through GCH1-dependent pathways, and restoring the coupling of eNOS to facilitate the availability of NO (105).

#### 5.7.3. Antihyperglycemic drugs

Endothelial dysfunction has been observed to be closely linked with hyperglycemia. Hence, the amelioration of hyperglycemia may lead to enhanced endothelial function. This can be accomplished through the utilization of various pharmacological agents, such as DPP-4 inhibitors, SGLT2 inhibitors, insulin, glucagon-like peptide-1 (GLP-1) receptor agonists, and metformin (106).

## 6. Role of MetS on hypertension

**Figure 4:**
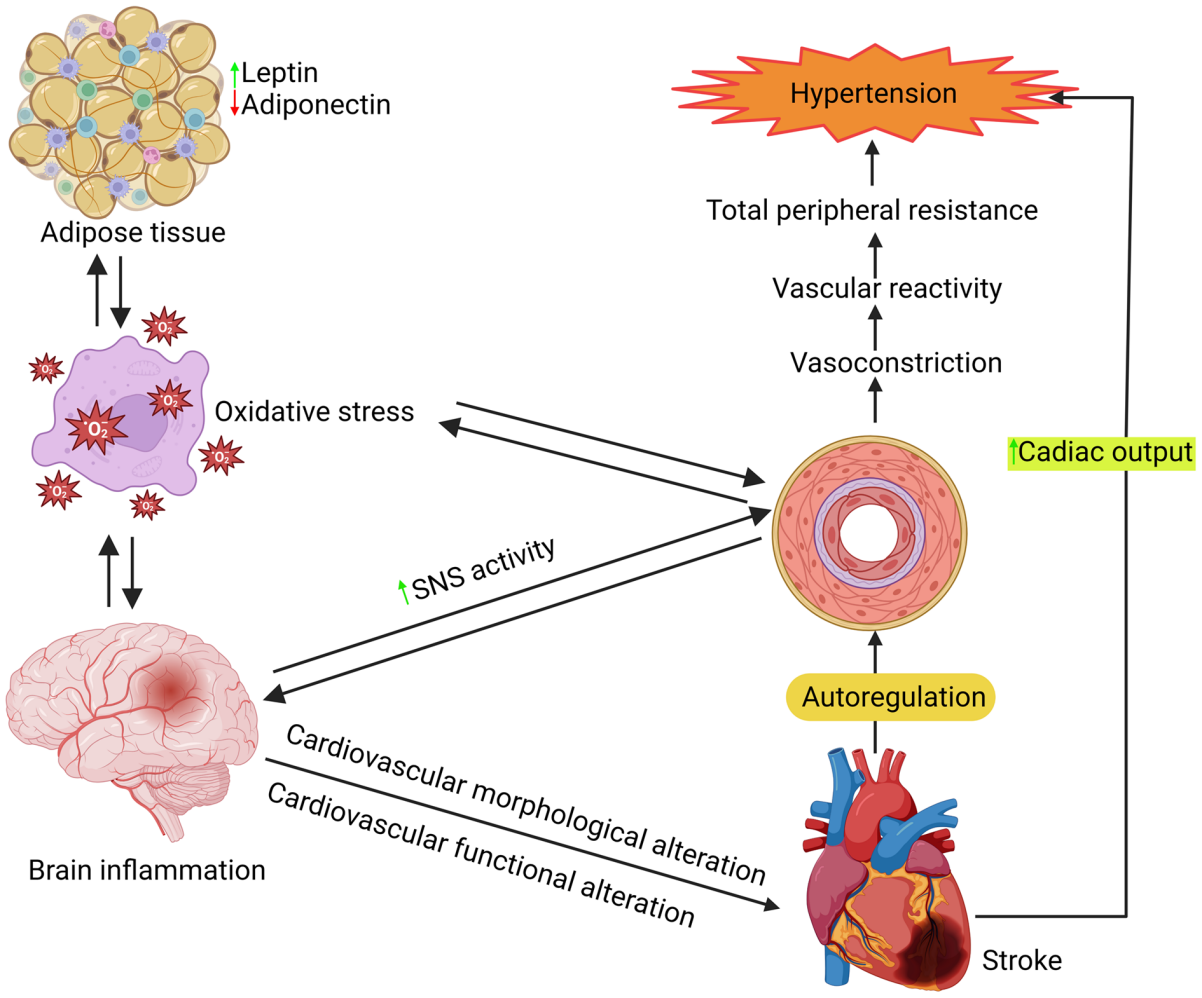
Illustration of a series of processes involved in the hypertension mechanism.

While there are various distinguishing aspects of MetS, hypertension is the most difficult to treat and is likely the least connected to or reliant on the condition (107). Hypertension affects around 80% of people with MetS. However, the majority of MetS patients with hypertension are overweight or obese. The most significant modifiable risk factor for CVD and overall mortality is hypertension, as shown in Figure 4. Hypertension is defined as systolic blood pressure (BP) of ≥140 mmHg or higher and diastolic blood pressure (BP) of ≥90 mmHg or lower, affecting 31.1% of the world’s adult population, or 1.38 billion people, in 2010 (108). Hypertension is rising internationally because of an aging population, increased exposure to lifestyle risk factors such as poor diets (high salt and low potassium consumption), and a lack of physical exercise. Globally, the prevalence of hypertension has fluctuated, although unevenly. Most hypertension has dropped somewhat in high-income countries during the previous two decades, while it has climbed dramatically in low- and middle-income nations.

### 6.1. Prevalence, prediction and geographical status of hypertension

Elevated blood pressure is a necessary criterion for diagnosing the syndrome. Research shows that hypertension is common in people with MetS (109). In the Pressioni Arteriose Monitorate E Loro Associazioni (PAMELA) study, high blood pressure was the most common component of MetS, affecting over 80% of patients. Individuals with MetS had higher blood pressure levels than those without the syndrome. Fatal events were more frequent in this population (110). Many hypertensive patients also have metabolic syndrome. In the Progetto Ipertensione Umbria Monitoraggio Ambulatoriale (PIUMA) study, 34% of hypertensive patients had MetS. Patients with MetS had more cardiovascular events than those without MetS (111). In a French study, MetS was increased with higher blood pressure values (112). In the PAMELA study, MetS-free participants were followed for 10 years. Individuals with certain types of hypertension were more likely to develop MetS (113). The Global Cardiometabolic Risk Profile in Patients with Hypertension Disease (GOOD) study found less than a third of hypertensive patients had acceptable blood pressure values. The study found that MetS was more common in participants with uncontrolled blood pressure than those with controlled blood pressure. The difference was significant (114). MetS in hypertensive patients increases end-organ damage. Hypertension and metabolic syndrome together lead to more cases of left ventricular hypertrophy, microalbuminuria, increased intima-media thickness, and hypertensive retinopathy (115). Hypertension has increased significantly over the past two decades in Southeast Asian and sub-Saharan African countries (116). The prevalence of hypertension and pre-hypertension among adults (18 years and older) in Uganda was determined through a cross-sectional survey representing the entire nation (117). The results showed an overall age- and sex-adjusted prevalence of 31.5% for hypertension and 38.8% for pre-hypertension. The majority of hypertension, adjusted for age and sex, was highest in the Central (34.3%), West (32.5%), and East (32.3%) regions. In comparison, it was lowest in the North (22.0%) and West Nile (24.1%) regions, based on geographical location (117). In the United States, hypertension is estimated to be prevalent when an individual has a systolic blood pressure of 140 mm Hg and a diastolic blood pressure of 90 mm Hg (118). The prevalence of hypertension in Southeast Asian urban populations was estimated at 33.82% based on pooled data. Among the total cases, 33.98% of hypertension was reported in the community, while 32.45% was reported among school adolescents. At present, at least 1 billion people worldwide suffer from hypertension. It is projected that this number will increase to 1.5 billion by the year 2025. A survey conducted in 2015 found that 25% of women and 20% of men suffer from hypertension. Less than 20% of people have well-controlled hypertension. Additionally, hypertension is associated with over 9 million deaths (116).

### 6.2. Oxidative stress and hypertension

There is a link between hypertension and increased oxidative stress in the vascular system; however, it is unclear whether oxidative stress is a cause or result of hypertension. It is well acknowledged that hypertension is the most critical risk factor for the development of CVD (119). A rising amount of evidence suggests that oxidative stress, which causes excessive ROS production, plays a significant role in the development of hypertension (120). ROS function as vasoconstriction mediators during vasomotor system modulation. Many factors, including angiotensin II, endothelin-1, and urotensin-II, trigger this vasoconstriction (121). In addition, the redox state influences the quantity of nitric oxide (NO) available for bioavailability in the body. NO is a potent vasodilator, and low levels of intracellular ROS play a crucial role in normal redox signaling that helps to maintain vascular function and integrity under physiological conditions (122). Higher levels of ROS contribute to vascular dysfunction and remodeling through oxidative damage when pathophysiological circumstances are present. Increased SOD and H_2_O_2_ generation, decreased nitric oxide synthesis, and impaired antioxidant bioavailability have all been associated with human hypertension (123).

### 6.3. Epigenetics and hypertension

It is now well-accepted that environmental and epigenetic, and genetic factors influence hypertension. Differential aetiologies of hypertension arise from complex interplays between genetic and environmental variables that modify biological pathways and lead to the disease (124). Hypertension significantly contributes to CVD pathologic remodeling, which may lead to severe complications such as heart failure, stroke, kidney failure, and cognitive impairment (125). Pulmonary vascular cells in people with progressive hypertension have a phenotype that is hyperproliferative, antiapoptotic, and inflammatory, leading to an increase in vasoconstriction and an aberrant remodeling of blood vessels. Gene mutations, epigenetic modifications, anomalies in sex hormones, and environmental variables all contribute to the development of hypertension (126). Altered DNA methylation patterns have been observed in patients with hypertension. Genomic DNA methylation has been associated with the onset and severity of hypertension (127). Individuals with chronic thromboembolic pulmonary hypertension exhibit differential methylation of multiple probes in pulmonary artery smooth muscle cells (PASMCs), which may be associated with PASMC remodeling. The epigenetic change in PASMC led to the activation of HIF-1, which caused a proliferative and antiapoptotic phenotype (hypoxia-inducible factor-1). It may be possible to differentiate between hypertension and pulmonary veno-occlusive diseases by analyzing dysregulated DNA methylation of specific genes (128).

### 6.4. Molecular mechanisms

It is crucial to comprehend the biology of hypertension to treat it and prevent any potential consequences effectively. Although there have been significant studies conducted on the pathophysiology and etiology of hypertension, it is still the case that over 95% of individuals with hypertension do not have a known cause. Vascular alterations, such as inflammation, remodeling, stiffness, calcification, and atherosclerosis, may play a role. Hypertension is a multifaceted condition that arises from the intricate interplay of genetic, physiological, and environmental factors. Hypertension’s pathogenesis has been linked to several variables, such as inflammation, renin-angiotensin-aldosterone system overexpression, sympathetic nervous system activation, and aberrant G protein-coupled receptor signaling, as shown in Figure 4 (129–131). A study indicates that changes in T-cell activity, specifically in the immune system, are a factor in the onset of hypertension (132). These processes commonly result in increased bioavailability of ROS due to excess ROS formation, decreased nitric oxide (NO) levels, and impaired antioxidant capacity in the arteries, heart, brain, and kidneys (133, 134).

ROS may cause hypertension by activating redox-sensitive signaling pathways. Maintaining a balance between NO production and ROS formation is crucial in the vasculature. Any NO generation reduction and ROS production increase might reduce blood flow. The functions of superoxide anion and H_2_O_2_ as second messengers are highly controlled. ROS stimulates the activity of many physiological components, including mitogen-activated protein kinases (MAPK), tyrosine kinases, Rho kinases, and transcription factors (NF-kB, AP-1, and HIF-1) and increased concentration of intracellular free Ca^2+^ also inactivates the protein tyrosine phosphatases (PTP). Furthermore, it increases the expression and activity of genes associated with inflammation and cancer (135, 136).

The pathophysiologic pathways in MetS connected to several diseases have been investigated. The objective is to find better and more effective therapies (137). So far, several distinct pathophysiological ideas have been proposed about why MetS patients have high blood pressure. The significant reasons are IR, obesity, activation of the sympathetic nervous system, and excessive salt consumption (138).IR raises heart rate and blood pressure via upregulating angiotensin II receptors and inhibiting nitric oxide synthesis (139). In addition, high levels of leptin, hypothalamic–pituitary–adrenal axis activity, obstructive sleep apnea, and baroreflex issues contribute to sympathetic nervous system activation in MetS (140). Finally, in obese patients, increased renal tubular reabsorption produces salt retention, which worsens high blood pressure. To summarize, the emergence of high blood pressure in patients with MetS is a multi-step process involving a variety of pathophysiological pathways (141).

### 6.5. Treatment

In cases where lifestyle modifications prove inadequate in achieving the desired blood pressure levels, pharmacological intervention may be deemed necessary. However, when hypertension continues after such therapy, antihypertensive medication is typically necessary, even for minor increases in blood pressure. The primary pharmacological agents utilized in the management of hypertension include diuretics, angiotensin-converting enzyme (ACE) inhibitors or angiotensin receptor blockers (ARBs), beta-blockers, and calcium channel blockers (CCBs) (142). Specific individuals may require multiple antihypertensive medications to attain their desired blood pressure level. In cases where blood pressure readings exceed the target by more than 20/10 mm Hg, newly diagnosed individuals may be promptly prescribed antihypertensive or a combination of hypertensive medications. To minimize adverse effects, taking a secondary medication with a complementary mode of action is advisable before taking the initial drug at the maximum recommended dose (143).

## 7. Role of MetS on platelet hyperactivity

**Figure 5:**
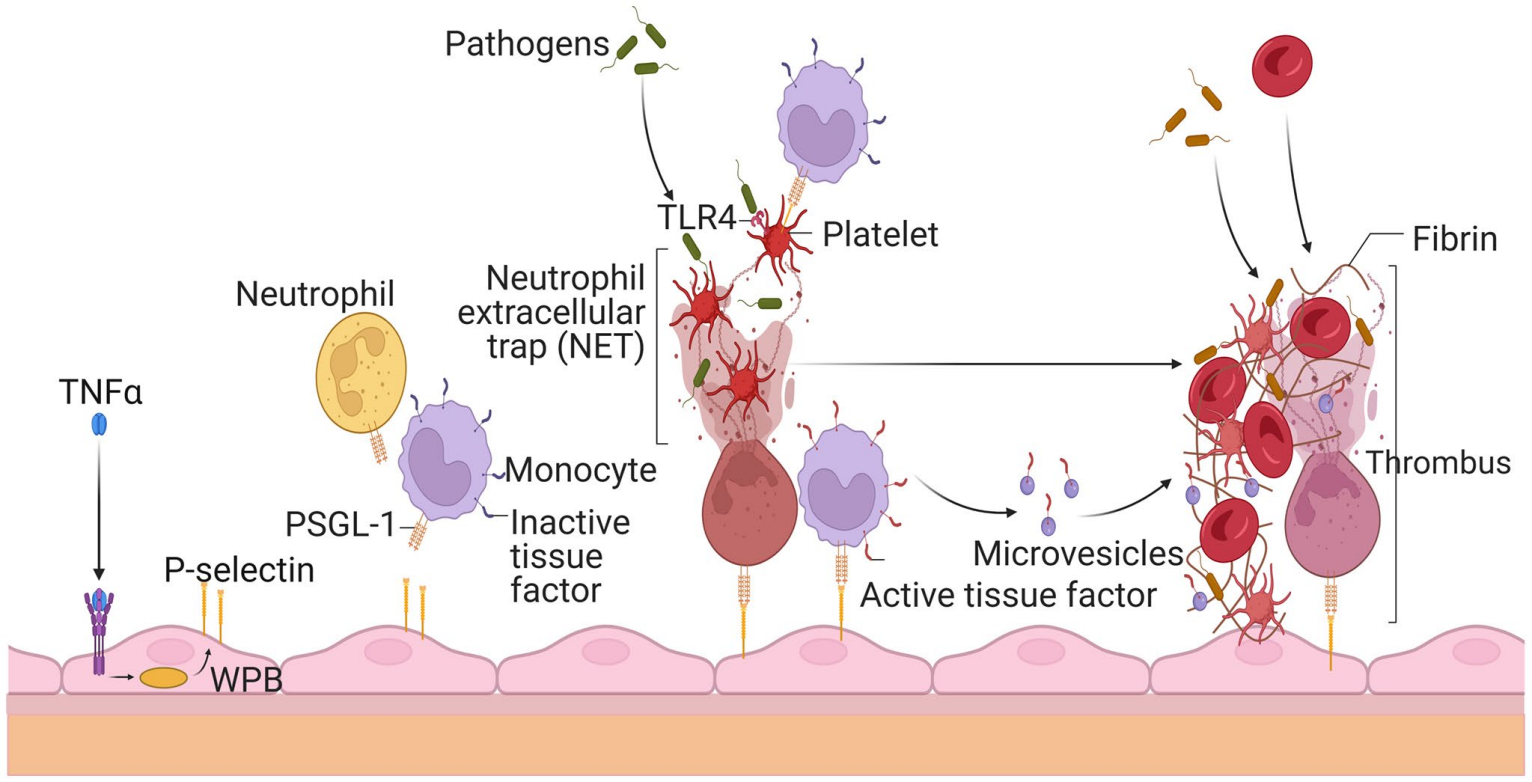
Thrombosis’s rapid spread due to circulating white blood cells and platelets. Endothelial cells release P-selectin through the exocytosis of Weibel Palade bodies (WPB) when stimulated by tumor necrosis factor alpha during inflammation. P-selectin glycoprotein ligand-1 (PSGL-1) recruits leukocytes such as neutrophils and monocytes by P-selectin. Neutrophil extracellular traps (NET) ensnare invaders, stimulate thrombus development, and activate platelets after being released by neutrophils. Platelets’ activation allows leukocytes to become activated and identify infections. Thrombus production is also encouraged by tissue factor (TF), which is released from the surface of monocytes and their microvesicles to initiate fibrin synthesis and red blood cell recruitment. The resultant thrombus aids in the entrapment of the infectious agent.

Platelets serve an essential role in hemostasis and thrombosis. Therefore, they should stay dormant and only become active when vascular damage occurs. When activated, platelets produce and release a slew of prothrombotic substances from their granules. Coagulation factor V, fibrinogen, and vWF are examples of these molecules (144). In inactivated platelets, several surface glycoproteins were expressed differentially (GP). During this activation phase, the adhesion molecule P-selectin is translocated to the cell surface and stored in endothelial cell Weibel Palade bodies and platelet granules (145). In addition, the GPIIb-IIIa on the surface of activated platelets changes conformation and binds to fibrinogen during activation. Platelets triggered by thrombin had decreased levels of vWF binding sites on the GPIb-IX complex but increased levels of GPIV critical to thrombospondin, shown in Figure 5 (146).

MetS patients often have hyperactive platelets. Elevated levels of fibrinogen, PAI-1, thrombin, von Willebrand factor, factor VII, and other coagulation factors, as well as significant platelet aggregation, define it (147). Specific abnormalities include high levels of tissue factor, factor VIII, fibrinogen, and inhibitor of plasminogen activator type 1 activity (148). At baseline, patients with MetS showed a lower antiplatelet response to aspirin and higher platelet reactivity. Platelet hyperactivity may be exacerbated by increased systemic inflammation, obesity, dyslipidemia, and other metabolic diseases (149). Platelet hyperactivity may also be exacerbated by metabolic diseases such as obesity. Increases in platelet count and mean volume (150) are one example of an abnormality associated with platelet reactivity. They are a prognostic sign in several atherothrombotic diseases, including acute coronary syndrome, thrombosis, and stroke.

Furthermore, an increase in cytosolic calcium concentration stimulates platelet reactivity (151). Finally, high blood leptin levels cause increased platelet aggregability. The overall effect of these anomalies is increased platelet adhesion and activation (152).

### 7.1. Prevalence, prediction, and geographical status of platelet hyperactivity

Platelet hyperactivity is becoming increasingly common worldwide, with its frequency and prevalence rising. It is the most prevalent type, comprising approximately 90–95% of all cases worldwide. The activation of these events results in an increase in vasoconstriction and the promotion of thrombus formation, which can ultimately lead to the development of atherosclerosis (16). No significant differences were observed between healthy participants with hyperactive platelets and those without hyperactive platelets regarding age, race, body mass index, smoking status, or the prevalence of hypertension. Several studies on humans have demonstrated a preference for platelet hyperreactivity among females (153). Platelet hyperreactivity is a common occurrence that can be triggered by different stimuli, indicating that similar platelet activation pathways are involved in the process. Platelet reactivity varies significantly among individuals and is associated with factors such as female gender, high plasma fibrinogen levels, and genetic diversity (154).

### 7.2. Oxidative stress and platelet hyperactivity

Thrombotic events are a common cause of morbidity and mortality in the elderly population. Much evidence suggests that oxidative stress regulates various elements of thrombotic processes, including platelet activation (155). Platelet or vascular redox state, endogenous or exogenous antioxidants, and the production of reactive oxygen and nitrogen species are all potential factors in platelet-dependent thrombus development (156). The parameters and processes that govern the synthesis and metabolism of superoxide and nitric oxide may alter platelet activity and thrombus formation (157). Normal platelet activation may cause a shift in the redox state. Glutathione disulfide levels rise, and oxygen consumption increases with platelet aggregation. Conditions that cause oxidative stress but do not cause a florid aggregation response have the potential to be prothrombotic (158). Platelets have essential roles in a wide range of biological processes, including but not limited to hemostasis, thrombosis, inflammation, infection, immunobiology, cancer metastasis, wound repair, and angiogenesis (159). Even though a significant change in the redox state occurs during normal aggregation; as a result, oxidative stress may promote platelet hyperactivity by reducing the level of physiologically accessible nitric oxide. Therefore, the measurement of oxidative stress may aid in the early diagnosis of asymptomatic patients at risk of developing thrombosis (160).

### 7.3. Epigenetics in platelet hyperactivity

Epigenetic changes may influence the generation and release into the circulation of proteins involved in blood coagulation and fibrinolysis. They may also interact with platelet activities and their susceptibility to antiplatelet medications (161). Cancer and other multifactorial illnesses, including atherosclerosis, hypertension, MetS, and diabetes, have received considerable attention during the last decade because of the potential relevance of epigenetic mechanisms in explaining the remaining unknown heredity factors. However, new research suggests that microRNAs and DNA methylation may be more nuanced in controlling the hemostatic balance, particularly in developing a prothrombotic condition linked to CAD (162). Platelet miRNA is involved in the regulation of hemostasis. Most microRNAs in human platelets are produced by megakaryocytes, which have an efficient miRNA processing mechanism (163). During thrombopoiesis, megakaryocytes provide platelets with most of their miRNA and pre-miRNA pools. Platelets may produce fully mature miRNAs (such as Dicer, Ago2, and RISC) expressing the enzymes necessary for pre-miRNA digestion. Ablation of Dicer 1 alters mRNA expression patterns and reduces miRNA expression levels in megakaryocytes and platelets (164).

### 7.4. Molecular mechanisms

The process of platelet activation is a complex phenomenon that is dependent on a multitude of factors. In the context of vascular injury, it has been observed that platelets possess adhesion receptors on their surface, namely integrins α6β1, α2β1, GPIIb/IIIa, and the GPIb/V/IX complex. These receptors exhibit specific binding interactions with laminin, collagen, fibrinogen, and VWF, respectively. Notably, this binding process occurs in the presence of regulatory molecules such as small G-protein regulators (SGRs), SRC-family kinases (SFKs), and serine/threonine-protein kinases (STKs). Consequently, these molecular interactions induce discernible changes in the morphology of platelets (165). When collagen binds to the platelet collagen receptors, it triggers a cascade regulated by phospholipase C (PLC). The depletion of calcium stores is a result of the thick tubular system. Activating various enzymes, such as phospholipase A2 (PLA2) and glycoprotein kinases, elevates intracellular calcium levels. This increase in calcium is crucial for several cellular processes, including morphological changes, presentation of the procoagulant surface, secretion of platelet granular content, and activation of Phospholipase A2 (166). The synthesis of thromboxane A2 from arachidonic acid, which serves as a precursor to TBXA2, is facilitated by the activation of phospholipase A2 (PLA2) and subsequent conversion by cyclooxygenase 1 (COX-1) (167). The G protein-coupled receptors that the released agonists activate include the thrombin receptor (F2R), thromboxane A2 receptor (TBXA2R), and adenosine diphosphate (ADP) receptors (P2RY1 and P2RY12). Upon adenosine diphosphate (ADP) activation, the P2RY12 receptor interacts with Gi and inhibits adenylate cyclase activity (168). The interaction of clotting factors with activated platelets is another vital process. The production of thrombin is triggered by the exposure of tissue factors in the arterial wall, which in turn sets off the clotting cascade. It plays a crucial role as a platelet activator by interacting with protease-activated platelet receptors (169). LAIR-1 and PECAM-1 are two proteins that are present in megakaryocytes. These proteins are crucial in keeping the platelets dormant within the bone marrow. In individuals with coronary heart disease, platelets are attracted to damaged blood vessels. Platelets assist in the process of blood clotting, thereby reducing bleeding. Contributing to the occlusion of sick arteries can worsen the condition, potentially leading to thrombosis (170).

### 7.5. Platelet hyperactivity and diabetes

Platelet hyperreactivity is a crucial determinant in developing thrombotic diseases such as heart attack and ischemic stroke, which may be fatal consequences of diabetes. Individuals with type 2 diabetes have higher amounts of the collagen receptor GPVI on the surface of their platelets (171). Elevated GPVI levels have been linked to cardiovascular events such as acute coronary syndrome, myocardial infarction, ischemic stroke, and transient ischemic episodes. Platelet activation is caused by the breakdown of atheromatous plaque or the de-endothelialization of the arterial wall. In order to conceal the exposed region, it is necessary to identify the sticky proteins through various receptors of platelet membrane glycoproteins (GPs) (172). When activated, a receptor triggers physiological reactions such as membrane phospholipid hydrolysis, intracellular calcium mobilization, and phosphorylation of vital intracellular proteins. The process of glycation of proteins on the surface of platelets has been reported to reduce membrane fluidity while boosting platelet adhesion. This, in turn, results in the incorporation of glycated proteins inside thrombi (173). Calcium mobilization from intracellular storage pools is associated with reduced membrane fluidity, which leads to increased intracellular calcium concentrations (174). Platelets in diabetic patients are hyperactive, with high adhesion, activation, and aggregation, which may be related to the dysregulation of various signaling pathways. Platelet activity was elevated in individuals with type 1 and type 2 diabetes in the early stages, which may increase their vulnerability to CVD (175). Platelet dysfunction in diabetics has the potential to be discovered before any visible damage to the vessel wall manifests itself. Diabetes increases platelet responsiveness to subthreshold stimuli, encouraging thrombotic events and the generation of new hyperreactive platelets (176).

### 7.6. Platelet hyperactivity and cardiovascular disease

Activated platelets are essential constituents of thrombi that obstruct arteries and contribute to plaque development in blood vessels during atherogenesis. Consequently, they are gaining prominence as novel participants, given that increased platelet aggregation constitutes a crucial risk factor for thrombosis, heart attacks, and strokes (177). The linkage between platelet activation and consequent thrombosis has been established in numerous CVD. The phenomenon of platelet hyperactivity encompasses many mechanisms, among which oxidative stress is included. Human platelets generate and discharge ROS, such as O^2−^, H_2_O_2_, or OH-, in reaction to physiological agonists. These ROS molecules play a crucial role in augmenting the platelet activation response through various signaling pathways, including isoprostane formation, Ca^2+^ mobilization, and NO inactivation. In addition, the generation of ROS by platelets, the absorption of free radicals from the environment, and the depletion of antioxidants can lead to pro-oxidant, pro-inflammatory, and platelet hyperaggregability effects, which increase the risk of CVD (178).

### 7.7. Platelet hyperactivity and infertility

Platelets are essential for hemostasis, and abnormalities in their activation or hyperaggregability may play a role in the pathophysiology of conditions, including miscarriage and thrombosis with no known cause. Miscarriage is increasingly connected to platelet hyperaggregability, also known as sticky platelet syndrome (SPS) (179). Hyperandrogenism, repeated anovulation, and infertility are hallmarks of polycystic ovary syndrome (PCOS), one of the most common endocrine disorders affecting women of reproductive age. Increased risk of CVD insulin resistance, and impaired endothelial function are all associated with this condition (180). Patients with PCOS exhibit increased agonist-induced platelet aggregation, faster coagulation, and microparticles produced from platelets. Factor XII deficiency, dysfibrinogenemias related to thrombosis, protein C defects, protein S defects, antithrombin deficiency, heparin cofactor II deficiency, and fibrinolytic defects associated with thrombosis are all linked to infertility and pregnancy loss. Pregnancy increases the risk of uteroplacental thrombosis in a hypercoagulable state because of coagulation factors, regulators, and fibrinolytic system changes. Furthermore, placental perfusion may cause thrombosis due to its low pressure and turbulent flow (181).

### 7.8. Treatment

Several therapeutic targets for platelet hyperactivity have been reported in studies aimed at reducing vascular risk in patients. Platelet-derived microparticles (PMPs) have been demonstrated to play a role in the increased incidence of atherosclerotic plaque and arterial thrombosis formation in individuals (182, 183). It is indisputable that patients with CVD exhibit platelets with a hyperactive phenotype characterized by higher aggregation, activation, and adhesion (184). This may contribute to the emergence of atherothrombotic complications in such patients (185). Hence, identifying elevated concentrations of particular microparticles can be a significant prognosticator of vascular impairment and cardiovascular consequences. New treatments have been explored using pharmacological drugs with antiplatelet and anticoagulant characteristics to improve human platelet function (186). For a long time, people have known that aspirin, or acetylsalicylic acid and cyclooxygenase (COX) inhibitor, has beneficial effects against inflammation and blood clots (187).

Over previous decades, it has been demonstrated that vitamin K antagonists possess anticoagulant characteristics. Dicoumarol, warfarin, phenprocoumon, acenocoumarol, and rivaroxaban are among the most significant vitamin K antagonists presently employed (188). Compared to aspirin, rivaroxaban reduced the risk of recurrence in patients with venous thromboembolism without significantly increasing the risk of bleeding (189). Clopidogrel, ticagrelor, prasugrel, and cangrelor are all examples of P2Y12 receptor antagonists, and each of these drugs has been shown to pharmacologically block adenosine diphosphate (ADP) receptors (186). Abciximab, tirofiban, and eptifibatide are examples of Glycoprotein IIb/IIIa inhibitors that have been shown to block fibrinogen adherence to activate platelets and, by extension, the formation of inter platelet bridges (190). The abovementioned medications treat and prevent atrial fibrillation and venous thromboembolism. Anticoagulants in question include unfractionated heparin and low molecular weight heparin. Fondaparinux, a parenteral anti-factor Xa medication, has lately emerged as a preferable alternative to unfractionated heparin (186).

## 8. Advanced/alternative treatment strategies

The primary goals of MetS treatment are to lower the risk of CVD and, ideally, to postpone the development of type 2 diabetes. Once type 2 diabetes has been established, medication may aid in maintaining health by reducing exposure to CVD risk factors (CVD). Therefore, the primary therapy for MetS is a lifestyle change that promotes cardiovascular wellness. Both nutritional supplements and physical exercise reduce MetS symptoms (191). Medicines, small molecules, and naturally occurring bioactive compounds are among the various materials that may be used in the treatment (192).

### 8.1. Pharmacotherapy

Because MetS is not yet a recognized disease and knowledge of common pathways as prospective therapy targets are still being researched, the current strategy is to address each problem individually. In addition to lowering the underlying risk factors, medication may be utilized to prevent CVD. Effective pharmacological therapies include statins for the treatment of dyslipidemia, antiplatelet medicines for the reduction of prothrombotic risk, and insulin sensitizers for the prevention of diabetes. There is no single pharmacological treatment for MetS, and patients with polypharmacy, limited adherence to current pharmacotherapy, and associated comorbidities find it challenging to continue taking multiple medications. Those who struggle to control their blood pressure are more likely to develop treatment-resistant hypertension. Antihypertensive drugs may now have unwanted side effects, an added complication. It has been suggested that aldosterone synthase, aldosterone receptors, and the ACE2/angiotensin 1-7/Mas receptor axis are all potential therapeutic targets (193).

Obesity is associated with type 2 diabetes, IR, and CVD, which may be avoided and treated using adiponectin, the principal peptide generated by adipocytes. Adiponectin levels are diminished by sickness. Endothelial, skeletal, cardiac, and adipocyte cells can produce this adipocytokine. Recently, the synthesis and release of higher-order adiponectin have been linked to several endoplasmic reticulums (ER)-associated proteins. These proteins include ER-resident protein 44 (ERp44), disulfide-bond A oxidase-like protein (DsbA-L), ER oxidoreductase 1- (Ero1-), and glucose-regulated protein 94 (GPR94). Adiponectin injections affect insulin sensitivity, atherosclerosis, inflammation, and weight in people and animals. Replacement treatment using human adiponectin may help identify therapeutic targets for preventing and treating metabolic diseases, including insulin resistance and type 2 diabetes (194).

Peroxisome proliferator-activated receptors (PPARs) regulate triglycerides (TGs), blood sugar, and abdominal adiposity. There are several kinds of PPARs. Fibrate and omega-3 fatty acid PPAR agonists lower TG. They raise HDL cholesterol via catabolizing TGs, especially when combined with fibrates (HDL-C). Glitazones, the major PPAR-agonist, decreases glucose but not TGs. Glitazones are antihyperglycemic and insulin-sensitizing. However, newer PPAR−/agonists such as elafibranor reduce TG while increasing HDL-C. They may aid in the treatment of NAFLD caused by MetS. As a result, the PPAR system can potentially treat atherogenic dyslipidemias; however, side effects such as myopathy, gallstones, and drug–drug interactions (such as those caused by gemfibrozil) must be considered (195). The indirect antioxidant effects of statins, type 1 angiotensin II receptor antagonists, angiotensin-converting enzyme (ACE) inhibitors, and other cardiovascular medications are pleiotropic. Effects on vasodilation, antithrombosis, and antiproliferation are facilitated by endothelial progenitor cells. The therapeutic advantages in heart failure might be attributed to these defense systems. The pathophysiological mechanisms that statins attack are identical. Statins lessen the risk of cardiovascular events, including heart attacks and strokes, by lowering inflammation and atherothrombosis in the blood vessels. Statins enhance NO bioavailability by decreasing NADPH oxidase activity in a Rac1-dependent manner, reducing caveolin-1 activity, decreasing asymmetric dimethyl-l-arginine, improving activating eNOS phosphorylation, and upregulating eNOS mRNA (196). Nebivolol, a third-generation beta-blocker, was shown to stimulate eNOS activity in *ex vivo* tests, leading to the induction of vascular nitric oxide, which explains why individuals with essential hypertension had better NO bioavailability (197, 198).

Subsequent research has revealed that while first- and second-generation beta-blockers do not inhibit Nox2, nebivolol does exhibit inhibitory effects on Nox2 in hypertensive rats and isolated cells (199). Directly modifying the cytoplasmic membrane assembly of Nox2 and cytosolic subunits p47phox, p67phox, and rac1 may result in various potential outcomes, including reducing superoxide generation, prevention of eNOS uncoupling and nitric oxide breakdown through interaction with superoxide, and enhancement of endothelial function. Furthermore, it has been observed that Nebivolol can mitigate oxidative stress in individuals with hypertension through the reduction of nitric oxide’s oxidative degradation (200).

### 8.2. Gene therapy

Gene therapy has progressed mainly to advances in delivery methods and vectors and the continual identification of novel mechanisms driving CVD (201). Clinical translation of cardiovascular applications lags substantially behind analogs for other conditions, such as metabolic diseases, blood disorders, and cancer. Differences in the intricacy and duration of the disease processes may account for this. In contrast to diseases resulting from a unique genetic mutation, most chronic cardiovascular conditions are multifactorial. The conditions mentioned above involve complex genetic and environmental interplays, rendering them less amenable to gene therapy (201). Attaining consistent expression in the cardiac or susceptible vascular tissue via vector delivery has presented novel obstacles. Adenoviral-mediated methods to overexpress sarcoplasmic-endoplasmic reticulum Ca2+/ATPase (SERCA) have shown promise in preclinical and early-phase clinical trials. The SERCA pump is a regulatory mechanism that significantly impacts the management of myocardial calcium homeostasis and contractility. This mechanism has been linked to heart failure (202). The phase II clinical study, Calcium Up-regulation by Percutaneous Administration of Gene Therapy in Cardiac Disease (CUPID2), yielded no significant therapeutic effects. This finding is documented in academic literature. It was hypothesized that AAV1-neutralizing antibodies were blamed for the virus’s failure to transduce SERCA. Disrupted redox signaling has not been the target of any gene therapy approach thus far. However, new targets from detailed molecular mechanistic analyses and improved gene therapy delivery technologies, such as using non-viral, nanoparticle-based DNA delivery mentioned below, provide renewed optimism.

It is well-established that vascular endothelial growth factor (VEGF) promotes angiogenesis. However, two additional benefits of improved redox balance are lowered NOX activity and eNOS coupling. VEGF may also stimulate NRF-2-mediated antioxidant mechanisms (184). The intramyocardial injection of adenoviral VEGF was safe and improved myocardial perfusion at 1 year in patients with severe CAD, as shown in phase I/II experiment. However, the precise distinction between increased redox signaling and direct activating pro-angiogenesis pathways remains to be seen (203).

### 8.3. miRNA therapy

MicroRNAs (miRNAs) are diminutive, non-coding RNA molecules that are crucial in regulating gene expression. MiRNAs exert significant control over translation through direct mRNA degradation and forming a protein complex called “RNA-induced silencing complex” (RISC), which can cleave miRNA targets. This underscores the critical role played by miRNAs in translation control. Both of these systems play a role in the regulation of translation (204). According to research findings, approximately 40 microRNAs have been observed to impact redox signaling, potentially in the onset of CVD or as a protective measure against it (205). In the last decade and a half, the discovery of AntagoMiRs, synthetic oligonucleotides with an anti-sense sequence explicitly targeting a particular miRNA, has been a significant breakthrough. Several side-chain modifications are also generated, making attaching to the target microRNA easier and providing protection from endogenous nucleases. These side-chain changes are also included. MicroRNAs, on the other hand, pose pharmacokinetic problems. They tend to amass in the kidneys and liver (206). Several nanoparticle compositions are now being developed to address these pharmacokinetic concerns. These formulations seek to increase antagoMir targeting to cardiovascular tissue while reducing cargo exposure to nuclease action. Currently, research on the cardiovascular system is only available in preclinical models.

In contrast, an antagoMiR that targets miR-34a has shown promise in a mouse model of heart attack by partly restoring myocardial function and boosting tissue survival rate. In addition, mir-34a increases oxidative damage in endothelial cells dependent on p66shc-sirtuin1. As a result, the benefits of antagoMiR-34a may be acquired by suppressing this apoptosis-promoting pathway (207).

### 8.4. Dietary strategies

Poor nutrition and lack of exercise are significant contributors to the worldwide rise in the prevalence of overweight and obesity. Until now, which dietary pattern is best for controlling MetS is unknown. Modifying one’s lifestyle, especially diet, is the most important therapeutic strategy for this condition. Certain dietary modifications have been demonstrated to effectively reduce symptoms and improve several metrics associated with MetS (184). Scientific studies showed that the DASH (Dietary Approaches to Stop Hypertension) diet is the most effective strategy for preventing and treating MetS compared to low-fat and restricted diets. Health practitioners may suggest simplified diets without restrictive diets. Normalizing metabolic imbalances in people with MetS requires calorie restriction and increased activity (208). MetSAs part of treatment for MetS; nutritional recommendations should consider the overall eating pattern since focusing on just one nutrient can only go so far. Alternatives to restrictive diets that focus on calories or specific nutrients have been found in recent research (209, 210). Numerous studies have shown the potential health advantages of following the MedDiet, including the primary and secondary prevention of CVD, T2DM, and MetS (211, 212).

The “Mediterranean Diet” describes the food preferences, lifestyle choices, and cooking methods of people living around the Mediterranean Sea region (213). The various plant-based foods include greens, fruits, grains, beans, nuts, seeds, olive oil, and pulses. Sofrito is a seasoning that has been traditionally utilized in various dishes. It comprises olive oil, tomato, garlic, onion, and leek, rich in phenolic compounds and carotenoids, including naringenin, hydroxy-tyrosyl, lycopene, and β-carotene (214). The traditional Mediterranean diet is characterized by a high-fat content and low carbohydrate intake, with fat contributing 35 to 45% of daily caloric intake, protein contributing 15%, and carbohydrates contributing 40 to 45%. The principal source of fat is derived from olive oil and almonds. The lipid in question is primarily comprised of polyunsaturated and monounsaturated fatty acids. Olive oil is the primary source of monounsaturated fatty acids (MUFAs) in the Mediterranean diet (MedDiet). In addition, olive oil is rich in oleic acid, which has been associated in several studies with beneficial effects on IR, blood lipid profiles, and blood pressure (215).

Olive oil is beneficial because of the polyphenols it contains, which have anti-inflammatory and antioxidant properties. Fruits and vegetables are rich sources of various nutrients, including antioxidant vitamins such as vitamins C, E and beta-carotene and phytochemicals, folates, and minerals (216). Recent studies have demonstrated that the Mediterranean diet (MedDiet) can reduce mortality rates in individuals of various body sizes, including those who are overweight or obese, physically fit, or at an increased risk for CVD (217). Studies have shown a negative correlation between MedDiet adherence and mortality from CVD, cancer, and other degenerative diseases. The risk of acquiring significant complications of CVD is also reduced by following the MedDiet, both statistically and clinically (218). Changes in body composition, as shown by lower total and segmental fat, may be related to differences in metabolic profile and other health outcomes associated with MedDiet treatment. The prevalence of both type 2 diabetes and CVD is falling, and research suggests that MedDiet may be contributing to this trend. Due to its many positive health effects, the MedDiet should be one of the first lines of defense in the fight against and management of MetS (219, 220).

There is a growing awareness of the significance of dietary factors in altering endothelial function. The primary focus of the research has been on n3 fatty acids, antioxidant vitamins (especially vitamins E and C), folic acid, and L-arginine (221). Studies have demonstrated that dietary factors significantly affect vascular reactivity. Studies have shown that fish oil and soy protein can improve endothelial function, which may help prevent CVD. These chemicals possess properties that protect the heart. Communities with a lower incidence of CVD have been given particular focus regarding their dietary habits. Research suggests that following a Mediterranean diet with a significant amount of vegetables, seafood, olive oil, and moderate wine consumption may positively impact endothelial function (222).

The risk of hypertension and its complications may be reduced by adopting dietary modifications that lower blood pressure. Nutritional therapies for hypertension prevention include reducing salt intake, limiting alcohol use, increasing potassium intake, and adopting a general healthy pattern like the DASH or Mediterranean diet (223). Recent research has shown that the DASH diet may effectively control hypertension. Consumption of fruits, vegetables, whole grains, low- or no-fat dairy, legumes, and nuts is preferred over red and processed meats and sugary drinks. The DASH diet is distinguished from other eating plans by its lower sodium intake (from 1,500 to 2,300 mg/day), higher fiber intake (>30 g/day), potassium, magnesium, and calcium content, and a lower intake of saturated fats (6% of energy) and dietary cholesterol (approximately 150 mg/d) (224). In epidemiological studies, improved adherence to the DASH diet has been related to a healthier cardiometabolic profile and a lower risk of CVD. The DASH diet’s food content and distribution are likely responsible for many health advantages. The DASH diet emphasizes their output, increasing potassium, magnesium, and fiber intake. Evidence shows that these nutrients help check blood pressure, glucose levels, and insulin sensitivity. Blood insulin and glucose levels have been shown to decrease in correlation with the consumption of polyphenol- and antioxidant-rich fruits and vegetables (225).

Avoiding or limiting the consumption of animal products is a central tenet of “plant-based diets.” In contrast, a focus on whole plant foods, including fruits, vegetables, nuts, legumes, and grains, is advocated. Consistent evidence links plant-based diets to a reduced risk of developing MetS and associated symptoms (226, 227). In addition, the risk of developing obesity, diabetes, and CVD, as well as the risk of dying prematurely, is lower among those who follow these dietary habits. In addition, the nutritional composition of plant-based diets promotes the consumption of a diverse range of plant-derived foods while discouraging the intake of red and processed meat, correlated with heightened susceptibility to diabetes, cardiovascular ailments, and certain forms of cancer. Furthermore, it has been established that the minerals and bioactive compounds in plant-based diets, such as vitamins C and E, beta-carotene, and polyphenols, exhibit antioxidant properties associated with reduced CVD and MetS (228, 229).

Several studies conducted in the last 20 years have demonstrated that the aqueous constituents present in tomatoes inhibit the aggregation of blood platelets, both *in vitro* and *in vivo*. The naturally-derived functional food component, Fruitflow®, a water-soluble tomato extract, has gained widespread global distribution and recognition (230). Fruitflow® is a dietary antiplatelet agent with a reversible mechanism of action and a milder antiplatelet effect than conventional antiplatelet medications such as aspirin, clopidogrel, and prasugrel (175). The antiplatelet tomato components present in Fruitflow® are known to protect the cardiovascular system by lowering platelet hyperactivity in response to potent platelet agonists such as adenosine diphosphate (ADP), collagen, arachidonic acid, thrombin, and inflammation. They may also aid in preventing endothelial dysfunction (231).

The findings of *in vitro* and *ex vivo* studies indicate that extracts derived from tomatoes and kiwifruits can mitigate platelet aggregation. The antiplatelet components found in tomato, strawberry, and kiwifruit have been observed to exhibit inhibitory effects on ACE (angiotensin-converting enzyme) and induce relaxation of the endothelium. These actions contribute to the protection of blood vessels (232). Recent studies have suggested that certain natural substances, such as olive oils, alperujo, ginseng, curcuminoids, and garlic, possess potential antiplatelet properties. Following the process of olive oil extraction, it has been observed that alperujo, the byproduct obtained, retains a significant amount of phenolic compounds. In their study, DeRoos et al. observed that applying alperujo extract at a concentration of 40 mg/L resulted in a notable reduction in platelet activation induced by ADP and TRAP in an *in vitro* setting. A recent study observed that the consumption of dietary vitamin-rich extra virgin olive oil for 1 year resulted in a significant reduction in ADP-induced blood platelet aggregation (233). The primary phenolic compound found in olive oil, known as hydroxytyrosol, has been observed to reduce platelet aggregation in humans (234) significantly. Ginseng has been historically employed to manage and mitigate cardiovascular disease (CVD) symptoms and its potential preventive properties. The inhibitory effects of ginsenosides on internal calcium mobilization and granule release have been observed, suggesting their potential as broad-spectrum antiplatelets (235). The platelet aggregation induced by ADP and arachidonic acid was significantly inhibited by curcuminoids derived from *Curcuma longa* plants (236). In a recent study, it has been observed that aged garlic extract can enhance the levels of cyclic nucleotides within the body. Moreover, it has been found to effectively inhibit the binding of fibrinogen, an essential protein involved in blood clotting. Additionally, the extract has been shown to mitigate platelet shape changes and modify their functional characteristics in response to collagen stimulation (237).

### 8.5. Natural bioactive compounds

Plant extracts, spices, herbs, and essential oils all include natural ingredients that have shown promise in treating MetS. However, these nutraceuticals are not suggested instead of standard pharmacotherapies for MetS since their advantages still need to be investigated (Table 1).

**Table 1:** List of Bioactive compounds and mechanisms in MetS.

**Bioactive compounds**	**Category**	**Source**	**Structure**	**Mechanism and effects**	**Reference**
Curcumin	Flavonoid polyphenol	*Curcuma longa* (Turmeric)	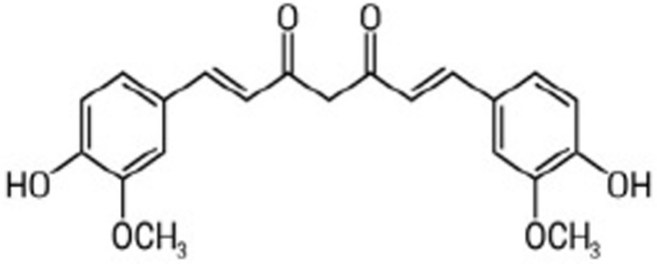	↑TNF-α, IL-6 ┴ NF-kB	(238)
Allicin	Vegetable	*Allium sativum* (Garlic)	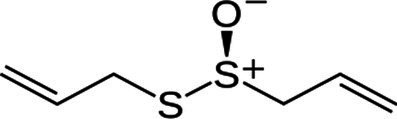	↑Nrf2 ↓SBP, Keap1, HIF-1α, and VEGF	(239)
Resveratrol	polyphenolic phytoalexin	3,5,4′-Trihydroxystilbene (Grapes, Apple)	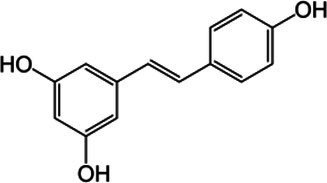	↑Nrf2, ↓NF-kB	(240)
Quercetin	Flavonoid	2-(3,4-Dihydroxyphenyl)-5,7-dihydroxy-4H-1-benzopyran-4-one. (Berries, Grapes, Onion)	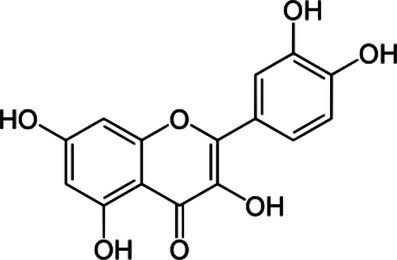	**↑**SOD, GPX, CAT ↓ROS, MDA, TNF-α, IL-1β, IL-10, Caspase-3, Bax/Bcl-2	(241)
Sulforaphane	Vegetable	1-Isothiocyanato-4-(methanesulfinyl) butane. (Broccoli, Cabbage)	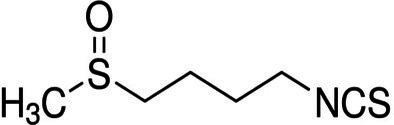	┴ Nrf2, NF-E2, NF-kB, MAPKs, P^38^, JNK, ERK ↓TNF-α, IL-6, IL-1β, PGE2	(242)
Cynaropicrin (Cardoon)	Vegetable	*Cynara cardunculus* (Artichoke thistle)	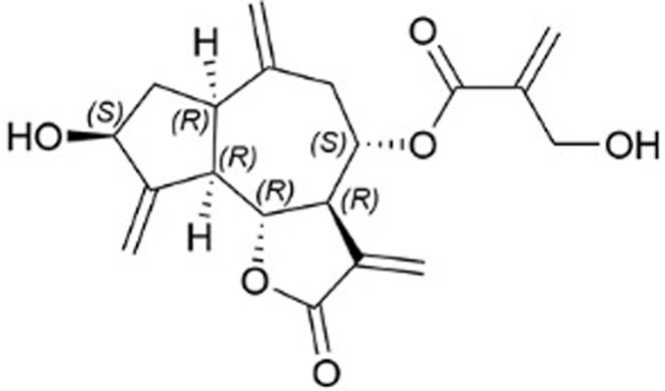	↓TNF-α, IL-6, ROS, NO, ERK ↑Nrf-2	(243)

#### 8.5.1. Curcumin

Curcumin is commonly found in Southeast Asian turmeric (*Curcuma longa*). Curcumin blocks NF-kB activation, resulting in lower levels of TNF-α and thrombotic plasminogen activator inhibitor type 1 (238). Furthermore, curcumin stimulates peroxisome proliferator-activated receptor gamma (PPR-γ) in hepatic stellate cells and blocks the Wnt/−catenin pathway, contributing to obesity. As a result of curcumin’s interference with leptin signaling, adiponectin expression is boosted. Obesity, IR, and inflammatory processes are all reduced in those with MetS (244). Research has shown that curcumin has the potential to enhance insulin sensitivity, inhibit the formation of fat cells, and lower high blood pressure, inflammation, and oxidative stress. As a result, it may help alleviate various aspects of MetS (245).

#### 8.5.2. Allicin

The anti-inflammatory and antithrombotic qualities of garlic/Allicin (*Allium sativum*) make it useful in medicine. According to a study, fructose-fed rats whose diet included raw garlic had greater insulin sensitivity, suggesting that this food item could have similar benefits for people (246). Research shows that a placebo-controlled study reduces total cholesterol and triglyceride levels (247). According to research, aged garlic extract altered MetS patients’ adiponectin levels and upregulated Nrf-2 express,sion, which raised adiponectin after 12 weeks (239). In addition, garlic’s organosulfur derivatives reduce inflammation. The garlic organosulfur compounds were found to effectively reduce dyslipidemia and liver fat by boosting taurine levels and encouraging hepatic fatty acid- oxidation which helps in maintaining healthy glucose levels due to its ability to inhibit DPP-4 and hepatic gluconeogenesis (248). Garlic can cure MetS naturally because its thiol groups fight ROS-mediated inflammation.

#### 8.5.3. Resveratrol

Resveratrol is a polyphenol found in grapes, nuts, and wine. It affects metabolism, oxidation, and aging via sirtuin pathway activation. It promotes lipolysis, reduces adipogenesis, and improves cellular energy balance. It inhibits antioxidant cyclooxygenase and Nrf-2 expression and downregulates NF-kB expression (240). Resveratrol enhances glucose tolerance, weight, insulin sensitivity, and BMI in MetS patients (249, 250). According to recent studies, resveratrol consumption has been linked to decreased risk of developing diabetes and its associated cardiovascular complications. Resveratrol has a protective effect due to its ability to regulate multiple signaling pathways. These pathways include those related to oxidative stress and inflammation, lipid metabolism, GLUT4 expression and translocation, and the SIRT1/AMPK signaling axis (251).

#### 8.5.4. Quercetin

Quercetin, a plant-derived flavonoid, is found in onions, berries, and tea. Its metabolic activities include antioxidant and anti-inflammatory. Studies have shown that quercetin may help reduce the risk of developing osteoporosis, CVD, neuropathy, and certain types of cancer (252). Furthermore, it possesses various other advantages such as antiatherosclerotic, anti-inflammatory, anti-obesity, anti-hyperlipidemic, anti-hypercholesterolemic, neuroprotective, anti-hypertensive, and anti-inflammatory properties (253). According to researchers, the metabolic effects of quercetin are thought to be influenced by the activation of transcription factors like PPAR-γ, AMPk, NF-kB, and SIRT1 (254). Quercetin inhibits obesity-causing adipokinesis and lipolysis through mitochondrial pathways. Obese Zucker fat rats were administered 2 mg/kg BW or 10 mg/kg BW of quercetin daily for 10 weeks. As a result, dyslipidemia, hypertension, and insulin resistance were reduced in these rats. The higher dose was the only one that effectively reduced body weight and inflammation in visceral adipose tissue. This was achieved by inhibiting the generation of tumor necrosis factor-alpha (255). Reduced adiponectin levels in the bloodstream have been linked to obesity, type 2 diabetes, and hypertension. Losing weight could lead to an improvement in these health conditions. The vasoprotective properties of quercetin have been linked to an increase in eNOS expression (255). Pfeuffer and coworkers discovered that in people with an apolipoprotein E genotype, quercetin reduces metabolic parameters, including waist circumference, postprandial blood glucose, and lipids, while increasing inflammatory markers like TNF-.α, IL-1β, IL-10, Caspase-3, Bax/Bcl-2 (241).

#### 8.5.5. Sulforaphane

The anti-inflammatory and antioxidant characteristics of a phytochemical called sulforaphane (SFN), found in the Brassica family of plants like broccoli, may be used to treat MetS. It induces NF-E2, Nrf-2, and NF-kB expression and decreases inflammatory markers like TNF-α, IL-6, and IL-1β (242). In addition, experimental animal research has shown that sulforaphane may prevent the development of MetS hallmarks such as diabetes, hypertension, and hyperlipidemia (256). SFN interacts with the active cysteine residues of KEAP1, dissociating KEAP1 and the subsequent activation of NRF-2 (257). SFN acts as a cytoprotective factor in different types of cellular stress by triggering the oxidative stress response (OSR) and decreasing inflammation. Administering SFN can help control the NF-kB pathway, which may prevent diabetic neuropathy and high glucose-induced changes in animals (258). SFN protects against diabetic cardiomyopathy through increased NRF2 expression and correction of oxidative stress-induced modulation of the LKB1/AMPK pathway (259). Mice with nonalcoholic fatty liver disease were administered SFN, reducing their oxidative stress, endoplasmic reticulum stress, and mitochondrial dysfunction (260). SFN reduces the expression of adipogenic factors such as PPAR and CCAAT/enhancer-binding protein (C/EBP), which inhibits adipocyte formation and fat accumulation. The detection of AMPK activation upon SFN administration suggests that SFN may have potential anti-obesity and anti-diabetic benefits (261). Supplementation with SFN decreased fasting blood glucose and glycated hemoglobin (HbA1c) levels in obese individuals with dysregulated type 2 diabetes. The anti-diabetic properties of this substance are similar to those of metformin, considered the standard medication for treating diabetes (262).

#### 8.5.6. Cardoon

The industrial potential of the cardoon plant is well acknowledged. It is an intriguing source of bioactive components, including cynaropicrin, phenolics, minerals, inulin, fiber, and sesquiterpene lactones, making it a functional food with great nutritional value. These bioactive compounds have anti-diabetic, anti-inflammatory, cardiotonic, antibacterial, anti-hemorrhoidal, antioxidant, anticancer, lipid-lowering, cytotoxic, and choleretic activities. The presence of vitamins, flavonoids, and polyphenols in *Cynara cardunculus L*. has been observed to contribute to its biological activity by scavenging free radicals and reducing oxidative stress. These properties have been associated with the plant’s hypolipidemic, hepatoprotective, and antidiabetic effects, as well as it’s capacity to increase Nrf-2 expression and reduce inflammatory markers such as TNF-α and interleukin-6 (243).

## 9. Conclusion

MetS is not classified as a disease *per se* but rather as a cluster of metabolic risk factors that have the potential to substantially elevate the likelihood of developing CVD. Despite several definitions for MetS, a singular purpose cannot be employed to assess potential hazards or outcomes in adolescents. Diagnosing the risk of MetS early on is imperative, as insulin resistance can lead to impaired glucose, lipid, and energy metabolism in various organs and tissues, resulting in the co-occurrence of multiple metabolic disorders. Alterations in migration, proliferation, and angiogenesis characterize the MetS. These modifications are caused by inflammation, hyperhomocysteinemia, oxidative stress, decreased angiogenic factors, and cellular senescence. Empirical data indicate a reciprocal association exists between hypertension, endothelial dysfunction, platelet hyperactivity, and MetS. The coexistence of hypertension, endothelial dysfunction, platelet hyperactivity, and MetS contributes to the advancement of organ damage. Thus, this population is critical to managing blood pressure, regulating the prothrombotic state, and controlling blood glucose.

A detailed understanding of the MetS mechanism will help develop effective prevention strategies and appropriate intervention tools. MetS and its components do not yet have specific therapy recommendations. Some patients are at moderate or high risk of developing atherosclerosis. The initial approach for managing the latter group involves implementing therapeutic lifestyle changes. In cases where the possibility of developing CVD is significant, it may be imperative to implement pharmacological measures to modify risk factors. Every person with MetS must go through a risk evaluation. Obesity, dyslipidemia, hypertension, insulin resistance, and hyperglycemia may all be successfully managed with dietary and physical activity changes. These treatments show the highest promise for managing MetS. A pharmacological approach targeting many MetS components and extensive gene and miRNA Therapy management may be necessary for high-risk people. MetS is often associated with gut microbiota dysbiosis, inducing a low-grade inflammatory response and insulin resistance via different mechanisms. This review did not discuss the gut microbiota as a potential target for treating MetS.

Although their success varies, lifestyle adjustments and other non-pharmaceutical methods are sometimes recognized as the first line of therapy. Improved insulin resistance, blood pressure, plasma lipid and lipoprotein metabolism, and general health may result from a diet high in nutrients and total calories, as well as from regular physical exercise and healthy body weight. Pharmaceutical therapy is only somewhat beneficial. In contrast to when lifestyle measures are used alone, it is connected with more significant adverse results when used in conjunction with them.

Nutraceuticals are a popular therapy for MetS due to their low toxicity and minimal adverse effects. Using curcumin, resveratrol, quercetin, aged garlic extract, sulforaphane, and fruitflow may be a natural treatment for hypertension, platelet hyperactivity, and endothelial dysfunction,. More efforts must be undertaken in animal and clinical studies to evaluate the influence of these compounds on CVD risk factors. In addition, the prevention and treatment of underlying risk factors might lower CVD prevalence in the general population.

## Author contributions

AD conceptualized the review. DD, GJ, and NRS wrote the manuscript. SP, AB, GJ, and AD corrected the manuscript. All authors have read and approved the final manuscript.

## Conflict of interest

The authors declare that the research was conducted in the absence of any commercial or financial relationships that could be construed as a potential conflict of interest.

## Publisher’s note

All claims expressed in this article are solely those of the authors and do not necessarily represent those of their affiliated organizations, or those of the publisher, the editors and the reviewers. Any product that may be evaluated in this article, or claim that may be made by its manufacturer, is not guaranteed or endorsed by the publisher.
